# A Novel Approach to Integrate Human Biomonitoring Data with Model Predicted Dietary Exposures: A Crop Protection Chemical Case Study Using Lambda-Cyhalothrin

**DOI:** 10.1021/acs.jafc.3c07071

**Published:** 2024-05-08

**Authors:** Nicholas Cuvelier, Raga Avanasi, Mark Grunenwald, Tharacad Ramanarayanan, Douglas C. Wolf, Scott M. Bartell

**Affiliations:** † Department of Environmental and Occupational Health, University of California, 856 Health Sciences Quad, Suite 3200, Irvine, California 92617, United States; ‡ Human Safety, Syngenta Crop Protection, LLC, P.O. Box 18300, Greensboro, North Carolina 27409, United States; § Department of Statistics and Department of Epidemiology and Biostatistics, University of California, Anteater Instruction Research Building, Suite 2030, Irvine, California 92697, United States; ∥ California Department of Public Health, 1631 Alhambra Blvd., Suite 200, Sacramento, California 95816, United States

**Keywords:** agricultural chemicals, National Health and Nutrition Examination Survey, exposure modeling, urinary biomarkers, pyrethroids, human health risk assessment

## Abstract

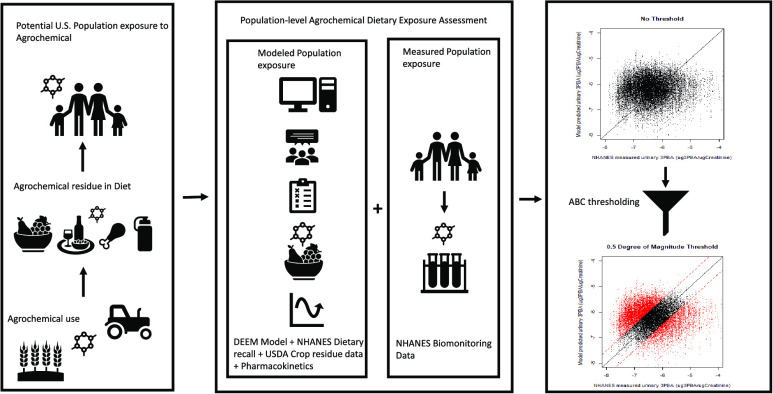

The appropriate use of human biomonitoring data to model population chemical exposures is challenging, especially for rapidly metabolized chemicals, such as agricultural chemicals. The objective of this study is to demonstrate a novel approach integrating model predicted dietary exposures and biomonitoring data to potentially inform regulatory risk assessments. We use lambda-cyhalothrin as a case study, and for the same representative U.S. population in the National Health and Nutrition Examination Survey (NHANES), an integrated exposure and pharmacokinetic model predicted exposures are calibrated to measurements of the urinary metabolite 3-phenoxybenzoic acid (3PBA), using an approximate Bayesian computing (ABC) methodology. We demonstrate that the correlation between modeled urinary 3PBA and the NHANES 3PBA measurements more than doubled as ABC thresholding narrowed the acceptable tolerance range for predicted versus observed urinary measurements. The median predicted urinary concentrations were closer to the median measured value using ABC than using current regulatory Monte Carlo methods.

## Introduction

Regulatory agencies such as the United States Environmental Protection Agency (US EPA) implement a robust regulatory framework, based on legislation, to protect the environment and humans through health-protective risk assessments.^1−6^ As part of this framework, regulatory agencies have increasingly looked for new ways to monitor potential pesticide or crop protection chemical exposure in human populations. Although potential exposure to pesticides can be measured by environmental monitoring (i.e., air, water, sediments, etc.), these approaches do not measure actual external exposures or internal concentrations of pesticides for individuals.^7^ Over the last few decades, biomonitoring has become an important tool to assess exposure to pesticides, providing a snapshot reflecting internal concentrations of specific pesticides in individuals at the exact time the biomarkers were collected, and potentially informing human health risk assessments and regulatory evaluations.^8−10^ Biomonitoring can be used to detect concentrations of specific pesticides in a variety of bodily fluids such as blood, urine, breast milk, or hair that result from integrated exposure across all exposure routes and pathways. The ability of human biomonitoring methods to potentially be both cost-effective and increasingly sensitive to low concentrations of chemicals identifies it as an extremely useful and powerful tool for pesticide exposure assessment.^11^

However, with a rise in the use of biomonitoring for assessing pesticide exposures, it is also important to recognize its limitations. Biomonitoring is often utilized in a cross-sectional manner, with one or few measurements for each individual, which can limit the interpretation of the results, especially when exposures are episodic or in cases of rapid clearance, causing substantial variation in biomarker concentrations over time.^12−14^ Additional issues to consider include the precision of biomarker measurements, the difficulties of separating variability over time from variability across individuals when repeated measurements over time for the same individuals are unavailable or scarce, and the still-developing understanding of the relationships between biomarker concentrations and potential health effects.^15−18^ Finally, biomarker data have mostly been collected for qualitative descriptive purposes and can be difficult to interpret and incorporate into quantitative risk assessments, though toxicokinetic modeling can facilitate this.^15,19^

Regardless of such limitations, the inherent advantage of biomonitoring data as a direct indicator of individual integrated dose has led to attempts to use such data in regulatory assessments.^9,20^ Due to the difficulties previously mentioned, these assessments have often relied on conservative assumptions to fill data gaps. For example, one recent risk assessment assumed a urinary excretion rate of 25% and steady-state pharmacokinetics in order to estimate a high-end population-level daily exposure rate from the 95th percentile of measured biomarker concentrations, ignoring the contributions of within-person temporal variation.^20^ When using biomonitoring data to estimate population-level pesticide exposure, perform risk assessments, and make regulatory decisions, a more robust statistical methodology that accounts for these complexities is warranted. In particular, when estimating population-level pesticide exposure from biomarker data, analysis should account for the episodic nature of dietary exposures, intra- and interindividual variability in behavioral factors driving exposure, and interindividuality of physiological parameters.^16,17,21−23^

The purpose of the study was to address this complex challenge of utilizing human pesticide biomonitoring data to meaningfully inform exposure assessments. We present a novel approach to integrate human biomonitoring data with regulatory model predicted population dietary pesticide exposures. Broadly, the present work builds on the U.S. EPA’s existing higher-tier approach in modeling population dietary exposures by adding an integrated Monte Carlo exposure, absorption, distribution, metabolism, and excretion (ADME) model to it, resulting in predictions of individual-level pesticide metabolite/biomarker concentrations. The utilization of biomonitoring data is then accomplished by a Bayesian calibration method via measured pesticide biomarkers used to account for temporal variation in exposure and account for key sources of parameter variability/uncertainty in the individual-level exposures.

Specifically, as a case study, we focus on dietary exposure to lambda-cyhalothrin and one of its metabolites, 3-phenoxybenzoic acid (3PBA). We started with the U.S. EPA’s existing higher-tier approach with the Dietary Exposure Evaluation Model (DEEM), which utilized individual-level dietary recall data from National Health and Nutrition Examination Survey (NHANES) and pesticide residue data from the Pesticide Data Program (PDP) to predict lambda-cyhalothrin dietary exposure estimates for the NHANES subpopulation that only included those individuals for whom 3PBA measurements were available.^24−26^ Our approach then combined the DEEM predicted individual dietary exposures with an ADME model (one-compartment pharmacokinetic model) to predict the urinary concentration of 3PBA (metabolized from lambda-cyhalothrin) for each individual at the time of recorded urine collection. Finally, the predicted urinary 3PBA concentrations were compared to measured urinary 3PBA concentrations for the same individuals using approximate Bayesian computing (ABC) to remove unrealistic exposure estimates outside of chosen acceptance thresholds. This approach is a significant improvement over existing approaches to integrate biomonitoring data with exposure assessment outputs, can be implemented as an easy add-on step to existing regulatory consumer exposure assessment methods, and can realistically inform crop protection chemical risk assessments and policy decisions.

## Methods

### Data Sets

The population used in this study includes individuals who participated in NHANES from 2007 to 2010. Although the DEEM software incorporates individual-level dietary data from NHANES 2005–2010, urinary 3PBA measurements are not available in the 2005–2006 cycle, so the data from participants in that cycle were excluded. For the 2007–2010 data sets utilized for this study, there were 5525 individuals who had laboratory work done with 348 missing 3PBA measurements. The differences in NHANES measured urinary 3PBA by demographic group were investigated, although only the participants that met the eligibility criteria were included (Table 1). NHANES uses a complex multistage probability sampling design to draw a nationally representative random sample of the U.S. population (excluding groups such as those institutionalized, members of the armed forces, citizens living abroad, and those living in nursing homes). NHANES is conducted by the National Center for Health Statistics (CDC NCHS), which operates as a branch of the Centers for Disease Control and Prevention (CDC). NHANES collects information about demographics, health, and nutrition while also performing physical examinations and laboratory measurements.

**Table 1:** Urinary 3PBA (ug/L) Percentiles Measured by Demographic NHANES 2007–2010 (for Those Meeting the Eligibility Criteria for Analysis)

demographic variables	25th percentile	50th percentile	75th percentile	90th percentile	95th percentile	sample size
total population	0.07	0.39	1.05	2.85	6.04	4211
males	0.07	0.4	1.06	2.77	5.69	2035
females	0.07	0.38	1.04	2.95	6.40	2176
age 6–11 years	0.07	0.4	1.15	3.33	7.59	578
age 12–19 years	0.07	0.36	0.85	2.34	4.95	620
age 20–59 years	0.07	0.42	1.09	3.22	6.53	1977
age 60+ years	0.07	0.36	0.98	2.42	5.40	1036
Mexican American	0.07	0.36	0.81	2.01	3.91	707
other Hispanic	0.07	0.44	1.25	3.02	5.03	383
non-Hispanic white	0.07	0.39	1.08	3.54	7.56	1792
non-Hispanic black	0.07	0.44	1.22	2.92	5.80	716
other incl. multiracial	0.13	0.47	1.13	2.56	5.042	178

NHANES conducts in-person dietary interviews with participants wherein participants report all foods consumed and mealtimes within the previous 24 h (midnight to midnight) on the day prior to the day they visit a mobile examination center (MEC). The dietary information in NHANES is collected via the What We Eat in America (WWEIA) dietary survey. The EPA developed the Food Commodity Intake Database to match recipes for each food listed in the WWEIA survey so that they can be broken down into raw agricultural commodities.^27^ These commodities can be matched to the PDP data to then determine the potential dietary exposure of a given person to a given pesticide based on the food they reported eating in the WWEIA survey. During the MEC visit, participants provide blood and urine samples, which were analyzed for various chemicals of interest including urinary 3PBA, which is a common metabolite of several pyrethroid pesticides. Participants were contacted again for a second 24 h dietary interview by telephone 3–10 days after the initial MEC visit. Both the dietary interview data and measured urinary metabolite concentrations were used to establish the PK model presented in this paper.

The United States Department of Agriculture (USDA) heads a food safety initiative known as the Pesticide Data Program (PDP).^28^ This program randomly samples fruit, vegetables, dairy, and other food types for pesticides and records residue levels for each food type. While participation in the program is not mandatory, there are around 600 sites throughout the U.S. that participate in the program (PDP). These residue data were used within the DEEM software to generate estimates of potential exposure to lambda-cyhalothrin through food ingestion with random sampling from among the different residue measurements for any particular crop within each Monte Carlo iterate.

### Population Dietary Exposure Modeling

**Figure 1:**
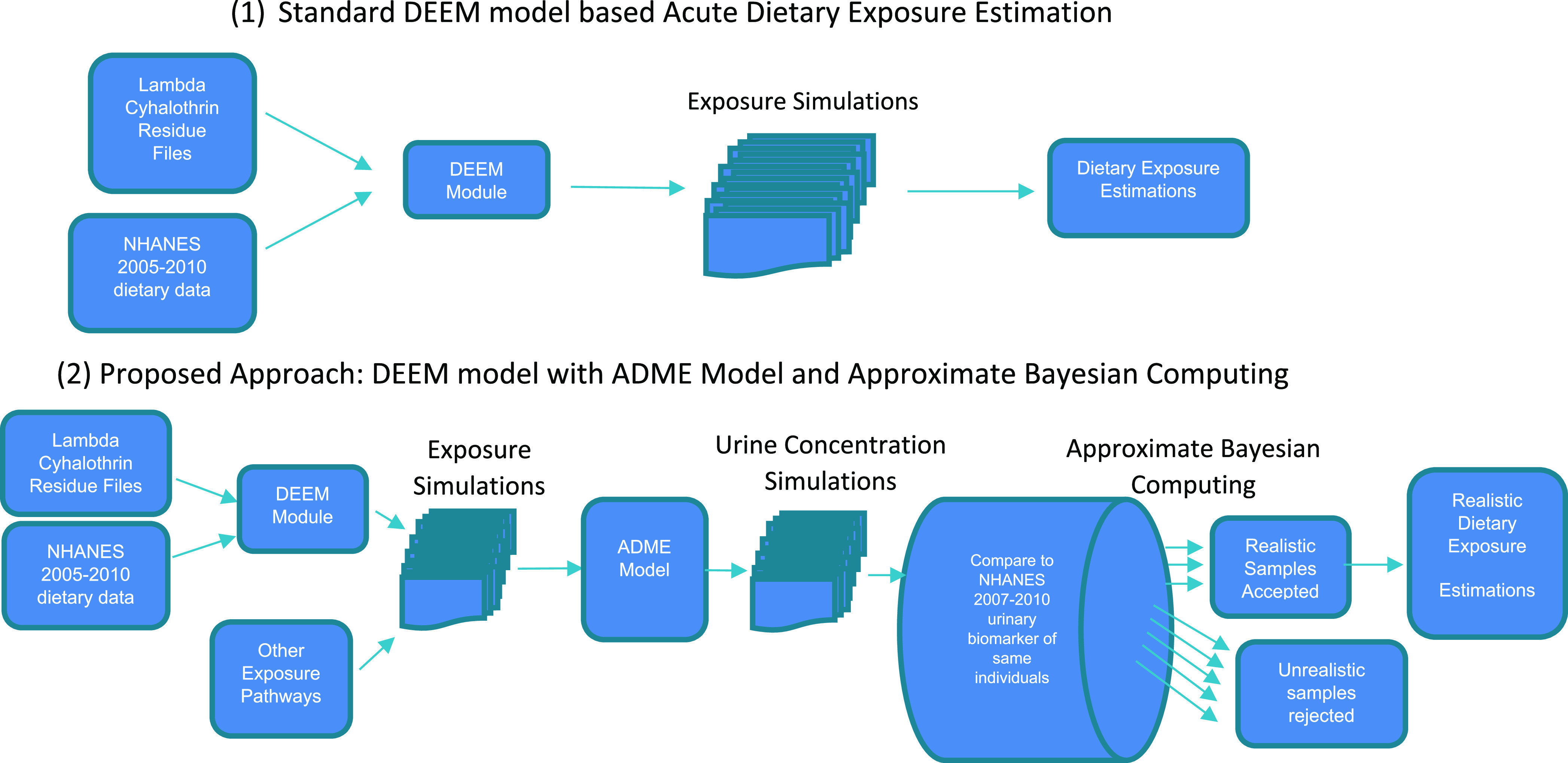
DEEM acute dietary exposure model Vs proposed approach.

The US EPA currently uses DEEM as the default regulatory model to estimate dietary (food and drinking water) exposures to the U.S. general population for use in pesticide risk assessments.^29^ DEEM-FCID version 4.02, commonly referred to as “DEEM,” is a dietary exposure model developed by Durango Software, LLC that is used to estimate exposure to pesticides in foods in the diets of the U.S. population. The software was based on food consumption data from the National Health and Nutrition Examination Survey (NHANES) and the What We Eat in America (WWEIA) survey. The DEEM model uses data from the NHANES participant dietary consumption and measured pesticide residue data (such as the PDP) to estimate dietary exposures to a pesticide (see panel 1 in Figure 1). In specific cases where matching NHANES participant’s biomonitoring data are available for a pesticide and/or their metabolites, our novel approach (see panel 2 in Figure 1) first combines the standard DEEM model-based exposure estimates with an ADME model to estimate internal biomarker concentrations of the pesticide and/or their metabolites. Next, an approximate Bayesian computing method is used to filter out unrealistic exposure estimates from the standard DEEM model outputs. Each of these models and methods is described in detail below.

### DEEM Exposure Estimates

We used the PDP pesticide residue data as input in the DEEM program to develop acute exposure estimates to lambda-cyhalothrin through food ingestion for individuals grouped into various subpopulations by age, replicating the U.S. EPA’s 2017 acute dietary exposure assessment.^30^ Using the pesticide residue data from PDP, crop residue files were created and matched to the ingredients of each food in the dietary intake data from NHANES participants from 2005–2010. The DEEM software uses the percent crop treated as well as the residue levels to develop exposure estimates. An acute assessment with Monte Carlo iterations was then run. The Monte Carlo iterations randomly assign a crop residue level appropriate for each food ingredient eaten by each individual and multiply the residue value by the amount of each food ingredient consumed. This is completed for each food ingredient, summed for all foods consumed for each individual on each day of 24-h dietary recall, and divided by that individual’s body weight to estimate the individual’s dietary exposure on that day. This procedure is repeated for each individual for the same number of times as the Monte Carlo iterations chosen by the user (5 for this demonstration), producing several plausible lambda-cyhalothrin exposure estimates for each individual for each day of the NHANES dietary recall (Figure 1).

DEEM is a standard model used by the US EPA in their regulatory risk assessments to estimate external dietary exposures for a representative U.S. population and does not have any capability to estimate internal exposures, i.e., predict any specific biomarker concentrations of pesticide and/or its metabolites. For this capability, we will need an ADME model that incorporates several individual participant-specific parameters, and our ADME model is described in detail below; a similar approach can be taken for other pesticides, provided necessary input data are available.

### ADME Model

The ADME model was built using the R Statistical software package, version 4.1.0, published by the R Foundation for Statistical Computing, using R libraries Rlab, nhanesA, zoo, dplyr, and chron and is based on previously published physiological parameters. We validated the model by comparing the results of predicted urinary measurements to the measured values reported in a separate controlled lambda-cyhalothrin oral dosing study with 6 volunteers with urinary 3PBA concentrations measured repeatedly for up to 120 h postdosing^31^ (see Supporting Materials).

After ingestion, lambda-cyhalothrin is rapidly metabolized to cis-3-(2-chloro-3,3,3-trifluoroprop-1-en-1-yl)-2,2 dimethylcyclopropanecarboxylic acid (CFMP), 3-phenoxybenzoic acid (3PBA), and other minor metabolites.^32,33^ Following the approach and data reported previously,^33^ a one-compartment pharmacokinetic model was modified to account for the distribution and urinary excretion of 3PBA, assuming that approximately 25% of ingested lambda-cyhalothrin is absorbed and quickly metabolized to 3PBA^31,33^ (Table 2), and accounting for mixing and storage in the bladder prior to urinary excretion. The final step is to standardize the urinary 3PBA concentration compared to the serum creatinine concentration. Demographic variables in NHANES were used to calculate clearance with formulas developed in previous studies for adults^34^ and for children (18 years or younger).^35^ With a piecewise constant dose rate (i.e., a constant lambda-cyhalothrin intake rate during any single hour, but potentially changing from hour to hour), the predicted serum 3PBA concentration *C*_*i,t*_ (in mg/L) for participant *i* at time *t* (in hours) is given by
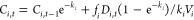
where *k*_*i*_ is the elimination rate constant for 3PBA for participant *i*, *f*_*i*_ is the fraction of ingested lambda-cyhalothrin that is absorbed and metabolized to 3PBA for participant *i*, *D*_*i*_*,_t_* is the ingested dose of lambda-cyhalothrin for participant *i* at time *t*, and *V*_*i*_ is the volume of distribution for 3PBA for participant *i*.

**Table 2:** Key Parameters for 3PBA Pharmacokinetics

parameter	description	point estimate	distribution
*k_i_*	elimination rate from plasma^33^	0.108 h^–1^	*t*_1/2_ = γ(24.2, 3.79)
			*k* = ln(2)/half-life
*f_i_*	percentage of lambda metabolized to 3PBA in serum^31,33^	25%	β(11.1, 31.4)
*V*_*i*_	volume of distribution^33^	17.7 L	γ(6.78, 0.38)
*R*_*i*_	percentage of urinary [3PBA + 4OH3PBA] as 3PBA^38^	58%	β(7.76, 5.86)
void	time between bladder voids^36,37^	5 h	{4,5,6}, with equal probability

Creatinine-standardized concentration of 3PBA in urine entering the bladder was modeled as *U*_*i*_*,_t_* (in μg of 3PBA per μg creatinine) for participant *i* at time *t* as
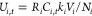
where *N_i_* is the creatinine excretion rate for participant *i*, estimated based on the individual’s body weight, height, age, and sex,^34^ and *R*_*i*_ is the percentage of urinary [3PBA + 4OH3PBA] as 3PBA for participant *i* (Table 2). Finally, the bladder storage delay and mixing is accounted for by averaging the urinary 3PBA concentrations entering the bladder over a period of 4–6 h before urination.^36,37^ Detailed descriptions of the parameter values for the dosing and pharmacokinetic model follow.

### Initial Conditions

Prior to the urine collection performed by NHANES, there is no direct information about previous serum or urinary concentrations of 3PBA for the participants. NHANES records 2 days of dietary information, though day 2 is conducted days after the urine collection, whereas day 1 consists of food consumption 24 h prior to the day of urine collection. In this model, exposure estimates based on day 1 and day 2 dietary information are averaged for each person and entered into the ADME model to generate a steady-state concentration of serum 3PBA for each participant. This steady-state concentration estimate is used as the initial condition at midnight on the first day of dietary recall, allowing for a realistic nonzero starting concentration of 3PBA in the body before dietary data are tracked.

### Hourly Dose Estimates on Day 1

Although there are 2 days of dietary data for each participant, only the dietary exposures occurring on the first day impact the urinary 3PBA concentrations measured for each participant because the urine samples were collected on the day after the first day of the dietary information (i.e., participants are asked at the time of urine sample collection to recall their diet for the previous day), and the second day of dietary information occurs at a later date. Due to the short half-life of lambda-cyhalothrin, ingestion on the day of urine collection could have an impact on 3PBA measurements, but no information about foods eaten was collected for the day of urine collection, only fasting times. To account for this, it was assumed that the meals on the day of urine collection were eaten at the same times reported by each participant for the previous day (day 1) and included the same foods and amounts as day 1. However, based on the length of fasting time prior to urine collection reported by each participant, meals that would have been skipped were excluded based on the reported fasting time. Because DEEM lists total daily exposure and does not disaggregate by meal, the assumption was made that the daily exposure to lambda-cyhalothrin was equally divided among all of the meals consumed by the participant on that day (i.e., the total exposure estimated from DEEM divided by the number of meals eaten that day), with each meal consumed during the hour self-reported by the participant for that meal time. Although some participants reported snacking at times other than meals, which contributed to the total estimated daily dose provided by DEEM, for simplicity, all food consumption was assumed to occur at the reported mealtimes. Further, any meals eaten on the day of urine sample collection were assumed to provide the same dose of lambda-cyhalothrin as each meal on the previous day.

### Food Washing Behavior

A food washing component was included in the model. A Food and Drug Administration (FDA) survey showed that 54–98% of respondents washed the queried fruits/vegetables prior to consumption.^39^ Different food washing/preparation techniques have been reported to reduce pesticide residues by 18–90%.^40^ A Bernoulli distribution was used to randomly assign each participant as either a “washer” or “non-washer” (washes food prior to eating or does not) for each Monte Carlo iterate, with 50% probability of being a food washer, set somewhat conservatively near the low end of the reported range. For washers only, the pesticide dosage was reduced by a washing factor or randomly selected from a β distribution centered at 30% reduction to reflect wide differences in the washing effectiveness depending on the method.

### ADME Parameters

The ADME model uses elimination half-life and volume of distribution parameters for 3PBA that were described previously.^33^ For Monte Carlo analysis, probability distributions (Table 2) were assigned to the 3PBA elimination rate (*k_i*), the percent 3PBA recovered in urine (percent_3PBA), and the volume of distribution (*V_i_*) based on previously reported means and standard deviations for these parameters.^31,33^ This allows for variation in these values centered around their respective point estimates to create a more realistic scenario in which these values are not identical for all individuals.

### Approximate Bayesian Computing

Finally, approximate Bayesian computing (ABC) was used to combine the Monte Carlo exposure estimates with the measured biomarker data. Briefly, Bayesian statistical methods can be used to combine “prior” information, such as DEEM lambda-cyhalothrin exposure estimates, with relevant measurements, such as urinary biomarkers, to obtain more realistic “posterior” estimates of the unknown individual exposure doses. Bayesian methods were proposed previously as a solution for integrating contact-based exposure estimates with measured biomarkers,^41,42^ but applications have been scarce due to the relatively complex algorithms and specialized software typically used for their implementation. In contrast, ABC offers many of the same advantages as fully Bayesian methods, but is simple to implement with a single added step within ordinary Monte Carlo simulation.^42,43^ The primary theoretical difference between ABC and fully Bayesian methods is that fully Bayesian approaches generate exact or near-exact posterior distributions under a given model and prior, whereas ABC relaxes the need for an explicit likelihood function, focusing instead on comparing prior Monte Carlo iterations to the measured values. The simplest form of ABC was used while discarding the Monte Carlo exposure iterates that are too discrepant from the corresponding biomarkers measurements to be plausible.^4^ For the ABC results, only exposure estimates producing predicted urinary 3PBA concentrations within thresholds of either an order of magnitude or half an order of magnitude from their respective measured values were accepted while rejecting the Monte Carlo exposure iterates that fell outside of those thresholds, i.e., accept the Monte Carlo iterate if the absolute value of the difference between log_10_ measured urinary 3PBA and log_10_ modeled urinary 3PBA is less than *c*, and reject otherwise, where *c* is 1 for an “order of magnitude” threshold and 0.5 for a “half an order of magnitude” threshold. “Unfiltered” results were compared with all Monte Carlo exposure iterates accepted regardless of the corresponding urinary biomarker measurements.

### Summary Statistics

Given the complex sampling design of the NHANES survey data, it is necessary to include the survey weights and design information when computing exposure percentiles to generate representative results. To do this, the results of multiple Monte Carlo iterations are averaged from each participant, and then the “survey” package in R was applied, which allows the inclusion of the pseudostratified primary sampling units (PSUs), the pseudostratum, as well as the MEC exam probability weights available in the NHANES data for each participant. Of note, since 4 years of NHANES data were combined (2 cycles), we constructed the weights by taking each MEC exam weight and dividing by 2 to account for the 2 cycles of data collection as recommended by NHANES. Although the weighted statistics changed slightly from the unweighted statistics, the overall differences between the measured and predicted data were largely similar. As indicators of model fit, correlations and residuals are presented using unweighted values.

## Results

**Figure 2:**
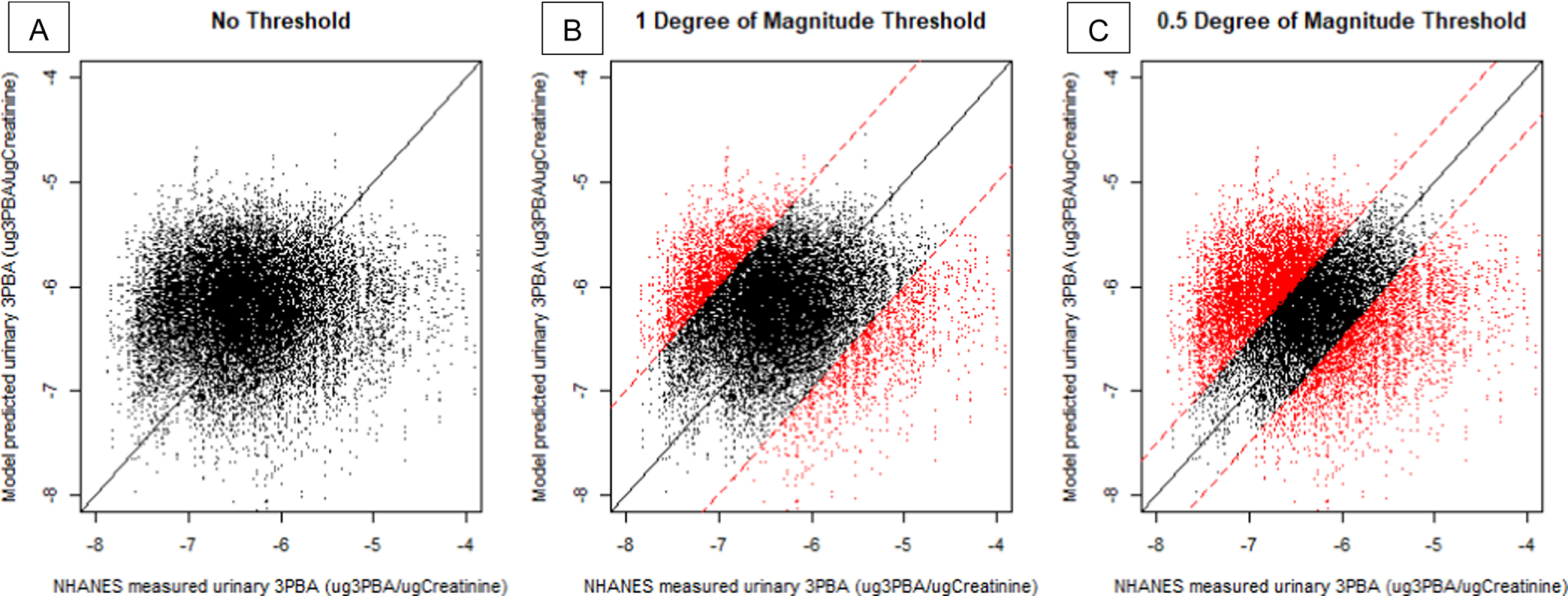
Predicted vs measured urinary 3PBA (log_10_ scale), without thresholding (A), rejecting model predictions more than 1 order of magnitude away from the measured values (B), and rejecting model predictions more than 0.5 orders of magnitude away from the measured values (C). In all panels, accepted model predictions are shown in black, and rejected model predictions are shown in red.

The present ADME model was validated by comparison to repeated measurements of urinary 3PBA concentration predictions over time in a controlled dosing study,^31^ resulting in adequately realistic predictions. Figure 2 compares the ADME model predictions of urinary 3PBA for NHANES participants to their measured urinary 3PBA concentrations, with Monte Carlo distributions to reflect parameter variability/uncertainty. Included are two separate accuracy thresholds for the model predicted urinary measurements (within either a full or half degree of magnitude above or below their respective measured values) to determine how this affects the correlation of the predicted and observed values and the survey-weighted percentiles for the exposure estimates. 80% of the model predicted urinary 3PBA values are within an order of magnitude of the NHANES measured values, and 48% are within a half order of magnitude, indicating that the exposure estimates and model predictions are largely reasonable compared to measured urinary concentrations (Supporting Figure 1). Although the unfiltered predicted values show a low correlation of 0.01 with individual measured values (*p* = 0.051), restricting the predicted measurements to those within a degree of magnitude of the NHANES measured values increases the correlation coefficient to 0.31 (*p* < 0.001), and the correlation coefficient is further increased to 0.69 (*p* < 0.001) using the half order of magnitude threshold.

ABC thresholding also changes the summary statistics for predicted urinary 3PBA concentrations, bringing the values closer to those of the original measured values in NHANES as thresholds become more restrictive (Figure 2). The median of the NHANES measured values is 3.5 × 10^–7^ μg-PBA/μg-creatinine. The median for the unfiltered model (panel A in Figure 2) predicted values was 7.1 × 10^–7^, but decreased to 6.8 × 10^–7^ and 6.3 × 10^–7^ for the slightly restrictive (panel B in Figure 2) and most restrictive (panel C in Figure 2) thresholds respectively. Finally, the original predicted lambda-cyhalothrin exposures from DEEM for the entire set of unfiltered Monte Carlo iterates to only those retained after ABC thresholding were compared. The original exposure predictions from DEEM had a median of 1.3 × 10^–4^ mg/kg/day and a 95th percentile of 3.4 × 10^–4^ mg/kg/day. The percentiles post-ABC thresholding had similar medians and 90th percentiles as the original unfiltered predictions, but showed progressively reduced predicted values of exposure at the 95th and 99th percentile with stricter ABC thresholding (Table 3). Similar patterns were observed for predicted urinary 3PBA concentrations, with reduced values for the upper percentiles with stricter thresholding (Supporting Table 1).

**Table 3:** DEEM Lambda-Cyhalothrin Exposure Estimates (mg/kg/day) before and after ABC

percentiles	original DEEM exposure estimates	1 degree of magnitude ABC	0.5 degree of magnitude ABC
median	1.3 × 10^–4^	1.4 × 10^–4^	1.4 × 10^–4^
90th percentile	2.8 × 10^–4^	2.8 × 10^–4^	2.8 × 10^–4^
95th percentile	3.4 × 10^–4^	3.4 × 10^–4^	3.3 × 10^–4^
99th percentile	4.9 × 10^–4^	4.8 × 10^–4^	4.7 × 10^–4^

## Discussion

### Strengths of the Proposed Approach

The present analysis demonstrated that reasonable estimates of urinary 3PBA concentrations can be obtained using dietary input from NHANES, crop residue files for lambda-cyhalothrin, and exposure estimates from DEEM in a literature-based ADME model. In addition, using ABC to restrict predicted values to within either a full degree or half a degree of magnitude above or below the NHANES measured values both increases the correlation of measured and predicted values while simultaneously bringing the median predicted values closer to the measured values. Finally, the closer the modeled urinary 3PBA concentrations were to the actual measured concentrations in the same individuals, the lower the estimated exposure to lambda-cyhalothrin was.

The ABC modeling approach is flexible, allowing for alternative model equations and parameters to account for differing ADME structures, multiple parent compounds and metabolites, or additional exposure routes without necessarily modifying the other model components or modifying the Bayesian implementation. Moreover, the ABC acceptance thresholds can be easily modified if desired, requiring predictions to be within any specified threshold for all measured metabolites. In the present example, the focus was on the primary exposure route of a common synthetic pyrethroid to demonstrate the method. In the future, characterizing exposure to all parent pesticides/chemicals of interest and measuring all their major metabolites could facilitate the effectiveness and accuracy of exposure and ADME models and better represent actual exposure to specific pesticides and other chemicals.

The ability to match DEEM data with NHANES data for both dietary data and urinary pesticide concentrations for the same individuals over 3 NHANES cycles serves as a powerful tool for studying a variety of exposures. In addition, this method could be used to better inform regulatory risk assessments for pesticides, for those pesticide-active ingredients with NHANES biomonitoring data of the parent and/or metabolite(s). Essentially, this approach could be a refinement tool, where if the traditional health-protective DEEM model exposure estimates result in any risks of concern, then NHANES biomonitoring (matched by participant) can potentially be used to compare measures and modeled dietary exposures, and after taking into account all of the assumptions and limitations of the modeling approach, it can be potentially used to inform policy decisions based on the risk assessment. This approach can also improve the understanding of the extent and types of input data needed for proper integrated dose reconstruction. While this has been presented as a method to estimate pesticide exposure, this same method could be applied to any other chemical monitored in NHANES and could incorporate both dietary and other routes of exposure.

### Limitations

While the model appears to produce reasonable results, there are potential limitations in both the inputs to the model and within the model implementation itself.

### Model and Data-Related Limitations

There are only 2 days of dietary data available for each participant, only 1 day of which was prior to the urine sample collection. Two days of data may not be sufficient as a measure of a person’s normal dietary habits and subsequent exposure to lambda-cyhalothrin, considering that within-person variability may be greater than between-person exposure.^13,17^ This is a limitation for several reasons: (1) For nonfasting participants, exposure on the day of urine collection could have a strong influence on the urinary 3PBA concentration later that day, (2) exposure on other days prior to urine sample collection could also have some impact, if unusually high dietary exposures occurred within a few days of urine collection, and (3) intrapersonal variability in dietary exposure could be a potentially large factor. For example, a dinner providing 5 times the usual daily dose of lambda-cyhalothrin would increase measured urinary 3PBA by about 10% 36 h later, but that dinner would likely not be captured by the 24 h dietary recall instrument used in NHANES or in DEEM, which only starts at midnight on the day before urine sample collection. Although missing dietary data were addressed with reasonable assumptions in this analysis (i.e., steady-state dietary exposure for prior days and identical mealtimes and exposure amounts for meals on the day of and the day before sample collection), prior meals and the baseline serum concentration are expected to have minimal impact on urinary excretion for most participants ∼36 h later. An additional limitation is a general lack of data regarding the impact of food washing, peeling/trimming, cooking, and other preparation behaviors on pesticide residue levels and dietary exposure.

DEEM output is limited to 40,000 lines. Given that all individual dose outputs to employ the ABC method are necessary, performing a large number of Monte Carlo iterations within a single run of DEEM for a large study population is not feasible. It is possible to perform multiple runs of DEEM under the same input conditions, merging results across runs to generate larger numbers of Monte Carlo iterations, but it is burdensome to combine large numbers of separate outputs. Thus, the NHANES populations were broken into subgroups, resulting in having to perform a low number of iterations for each subgroup, and finally merge all of the outputs back together to form Monte Carlo results with 5 iterations for the entire NHANES population. Considering the superior capabilities of modern computers and operating systems, this limit on the output file size seems to be an arbitrary software limitation that could be remedied in future versions of DEEM or through a modern dietary modeling platform like Cumulative and Aggregate Risk Evaluation System–Next Generation (CARES–NG).^44^ Until then, it is possible to repeat the DEEM runs as many times as desired, combining multiple output files to obtain the desired number of iterations.

### Implementation Limitations

The present pharmacokinetic model functions to predict urinary 3PBA concentrations after estimating lambda-cyhalothrin exposure based on dietary data. 3PBA is not only a metabolite of lambda-cyhalothrin but a metabolite common to many other pyrethroid insecticides. Currently, 3PBA is the only lambda-cyhalothrin metabolite measured in NHANES. The present model was not able to track multiple different parent compounds or multiple metabolites; thus, it was assumed that all of the measured 3PBA originated from lambda-cyhalothrin. Attributing all urinary 3PBA to lambda-cyhalothrin did not account for the contribution of other pyrethroids that are metabolized to 3PBA, likely underestimating total pyrethroid exposure and overestimating exposure to lambda-cyhalothrin. Had other parent compounds been included in the exposure model, the unfiltered urinary 3PBA predictions would have been higher, resulting in preferential selection during the ABC step of Monte Carlo iterates for which lambda-cyhalothrin explained a smaller proportion of measured urinary 3PBA, rather than most or all of it. This limitation was determined to be acceptable in the present case as it results in a highly health-protective exposure determination by assuming only one source of the metabolite. Also, the PDP measurements used for the present model did not distinguish between γ and lambda-cyhalothrin and instead measured “total cyhalothrins” which could include both compounds. This is a reasonable assumption because the estimated usage of γ cyhalothrin in 2018 was approximately 1% that of lambda-cyhalothrin usage.^45^ In addition, the co-occurrence of multiple pyrethroids in the same food item and coexposure to multiple pyrethroids in the same day may be less probable. Szarka and Ramanarayanan^46^ analyzed the co-occurrence of conazole fungicides in food commodities reported by PDP and found that the probability of presence of multiple conazoles in single food commodity samples is below 2%. Future work may include a similar assessment of co-occurrence of pyrethroid residues in food commodities reported by PDP.

The present study focused solely on dietary exposure to lambda-cyhalothrin, but other exposure routes could also be important. For example, residential exposure is not specifically evaluated within NHANES, but there are recommendations made by the EPA based on conservative models for residential exposure values.^47^ While it was attempted to include these residential exposure recommendations in the present modeling effort, the resulting urinary 3PBA predictions that included both dietary and residential exposure to lambda-cyhalothrin proved highly unrealistic, as they were several orders of magnitude greater than the values actually measured in NHANES. Since the data did not support addition of residential exposure to the model, this additional exposure route was not included. These results also point to the need for more realistic measures of residential exposure to pesticides.

Additionally, this analysis relies on a simple one-compartment model. More sophisticated multicompartment models could be created that would account for additional bodily compartments and transfer rates. However, as shown in the Supporting Materials, a two-compartment model did not necessarily result in more accurate biomarker predictions. The one-compartment model presented here appears to be reasonably accurate and sufficient for this application.

Monte Carlo simulations were used with repeated draws from the same population with some participants excluded based on data availability and ABC thresholding. This resulted in an issue with the normal method of applying NHANES weights to the analysis, which are computed assuming all MEC participants are included in the results exactly once. However, the main interest of this study is to demonstrate and understand the impact of applying ABC methodology within an integrated exposure and dose modeling system and comparing it to ordinary Monte Carlo analysis. Therefore, we felt that averaging filtered or unfiltered Monte Carlo iterations across each included participant prior to employing the survey weights was acceptable and consistent with other NHANES analyses with minor missing data exclusions. As a sensitivity analysis, we also tested applying the weights without accounting for multiple draws of the same person or loss of participants via missing variables or thresholding, and there was very little (<5%) change in the results, though this is not the most appropriate way to conduct weighted analysis.

### Lessons for Future Work

It is useful to note that the results of this study speak to the importance of data availability in biomonitoring studies. First, it is important to analyze the half-life of the chemical of interest in the body and include repeated measurements over time in order to inform time-dependent models. Also, if biomarkers are measured in urine, measuring and reporting on creatinine would also be of value. This would allow researchers to take interindividual variability in urine volume into account by utilizing creatinine correction for comparisons between individuals. Finally, collecting detailed information regarding the different exposure factors by the anticipated exposure routes such as dietary information (types, amounts, and timing) both the day before and the day of biomarker collection would be useful as well, considering the impacts of recent exposures on measured biomarkers.

The presented model, despite inherent limitations, reasonably predicts urinary concentrations of 3PBA based on estimated lambda-cyhalothrin ingestion from dietary recall. The ability to combine biomarker data from NHANES participants with exposure model results (for the same set of NHANES participants) from DEEM serves as a powerful tool for predicting pesticide metabolites in urine and for refining Monte Carlo exposure estimates and could potentially be used for various other chemicals. Previous assessments have relied on very conservative assumptions and overly simplistic steady-state models to interpret biomarker data; ABC and similar approaches can improve the accuracy of Monte Carlo exposure simulation and provide more insights into population pesticide exposures.

## Data Availability

The data sets analyzed during the current study are available in the USDA PDP repository, https://www.ams.usda.gov/datasets/pdp/pdpdata, and the CDC NHANES repository, https://wwwn.cdc.gov/nchs/nhanes/.

## Supporting Information Available

Supporting materials: validation of ADME model, Table S1 showing model predicted vs measured urinary 3PBA values (μg 3PBA/μg creatinine) with and without thresholding, Figure S1 showing a histogram of urinary 3PBA residuals (log Predicted–log Observed) with and without ABC thresholding, and R code (Supplemental R code S1–S6) (PDF)

## Abbreviations

3PBA: 3-phenoxybenzoic acid
ABC: approximate Bayesian computing
ADME: absorption, distribution, metabolism, and excretion
CARES–NG: Cumulative and Aggregate Risk Evaluation System–Next Generation
CDC: Centers for Disease Control and Prevention
CFMP: cis-3-(2-chloro-3,3,3-trifluoroprop-1-en-1-yl)-2,2 dimethylcyclopropanecarboxylic acid
DEEM: Dietary Exposure Evaluation Model
FDA: Food and Drug Administration
LOAEL: lowest observed adverse effect level
MEC: mobile examination survey
NCHS: National Center for Health Statistics
NHANES: The National Health and Nutrition Examination Survey
PDP: Pesticide Data Program
PSU: primary sampling units
USDA: The United States Department of Agriculture
US EPA: United States Environmental Protection Agency
WWEIA: What We Eat In America

## References

[ref1] Alavanja M. C. R. Introduction: Pesticides use and exposure extensive worldwide. Rev. Environ. Health 2009, 24 (4), 303–309. 10.1515/REVEH.2009.24.4.303. 20384038 PMC2946087

[ref2] Atwood D.; Paisley-Jones C. Pesticide Industry Sales and Usage 2008–2012 Market Estimates, 2017.

[ref3] USDA-ERS. USDA ERS - 2019 Data Overview, 2019. https://www.ers.usda.gov/data-products/agricultural-trade-multipliers/2014-data-overview/.

[ref4] United States Environmental Protection Agency. Overview of Risk Assessment in the Pesticide Program. https://www.epa.gov/pesticide-science-and-assessing-pesticide-risks/overview-risk-assessment-pesticide-program (accessed February 20, 2024).

[ref5] Moore D.; McCarroll-Butler C.; Avanasi R.; Chen W.; White M.; Brain R. How Protective to the Environment is the Pesticide Risk Assessment and Registration Process in the United States?. J. Regul. Sci. 2021, 9 (2), 1–20. 10.21423/JRS-V09I2MOORE.

[ref6] Avanasi R.; Glover A.; Lord C.; Macari J.; Munday M.; McKillican C.; Algarin N.; McCaskill A.; Hampton R.; Brain R.; Leiner K. How Protective is the Pesticide Risk Assessment and Registration Process to Humans in the United States?. J. Regul. Sci. 2023, 11 (1), 1–29. 10.21423/JRS.REGSCI.111249.

[ref7] Justino C. I. L.; Duarte A. C.; Rocha-Santos T. A. P. Recent progress in biosensors for environmental monitoring: A review. Sensors 2017, 17 (12), 2918 10.3390/s17122918. 29244756 PMC5750672

[ref8] Ross J.; Chester G.; Driver J.; et al. Comparative evaluation of absorbed dose estimates derived from passive dosimetry measurements to those derived from biological monitoring: Validation of exposure monitoring methodologies. J. Exposure Sci. Environ. Epidemiol. 2008, 18 (2), 211–230. 10.1038/sj.jes.7500591. 17593947

[ref9] U.S. Environmental Protection Agency G of U. Guidelines for Human Exposure Assessment Guidelines for Human Exposure Assessment. US Environ Prot Agency, 2019. https://www.epa.gov/risk/guidelines-human-exposure-assessment.

[ref10] Sobus J.; Marsha M.; Joachim P.; Barr D. Biomonitoring: Uses and Considerations for Assessing Non-Occupational Human Exposure to Pesticides, 2010.

[ref11] Angerer J.; Ewers U.; Wilhelm M. Human biomonitoring: State of the art. Int. J. Hyg. Environ. Health 2007, 210 (3–4), 201–228. 10.1016/j.ijheh.2007.01.024. 17376741

[ref12] Bartell S. M.; Griffith W. C.; Faustman E. M. Temporal error in biomarker-based mean exposure estimates for individuals. J. Exposure Anal. Environ. Epidemiol. 2004, 14 (2), 173–179. 10.1038/sj.jea.7500311. 15014548

[ref13] Attfield K. R.; Hughes M. D.; Spengler J. D.; Lu C. Within- and between-child variation in repeated urinary pesticide metabolite measurements over a 1-year period. Environ. Health Perspect. 2014, 122 (2), 201–206. 10.1289/ehp.1306737. 24325925 PMC3915262

[ref14] Li A. J.; Martinez-Moral M. P.; Kannan K. Temporal variability in urinary pesticide concentrations in repeated-spot and first-morning-void samples and its association with oxidative stress in healthy individuals. Environ. Int. 2019, 130, 104904 10.1016/j.envint.2019.104904. 31226556 PMC6682452

[ref15] Hays S. M.; Aylward L. L.; LaKind J. S.; et al. Guidelines for the derivation of Biomonitoring Equivalents: Report from the Biomonitoring Equivalents Expert Workshop. Regul. Toxicol. Pharmacol. 2008, 51 (3 SUPPL.), 4–15. 10.1016/j.yrtph.2008.05.004. 18583008

[ref16] Tan Y. M.; Dary C. C.; Chang E. M. Biomonitoring–An Exposure Science Tool for Exposure and Risk Assessment; US Environ Prot Agency: Washington, DC, USA, 2012.

[ref17] Egeghy P. P.; Cohen Hubal E. A.; Tulve N. S.; et al. Review of pesticide urinary biomarker measurements from selected US EPA children’s observational exposure studies. Int. J. Environ. Res. Public Health 2011, 8 (5), 1727–1754. 10.3390/ijerph8051727. 21655147 PMC3108137

[ref18] Calafat A. M. Contemporary issues in exposure assessment using biomonitoring. Curr. Epidemiol. Rep. 2016, 3 (2), 145–153. 10.1007/s40471-016-0075-7. 28884070 PMC5584386

[ref19] Scher D. P.; Sawchuk R. J.; Alexander B. H.; Adgate J. L. Estimating absorbed dose of pesticides in a field setting using biomonitoring data and pharmacokinetic models. J. Toxicol. Environ. Health, Part A 2008, 71 (6), 373–383. 10.1080/15287390701801638. 18246497

[ref20] Pest Management Regulatory Agency. Lambda-Cyhalothrin, 2017.

[ref21] Avanasi R.; Shin H. M.; Vieira V. M.; Bartell S. M. Variability and epistemic uncertainty in water ingestion rates and pharmacokinetic parameters, and impact on the association between perfluorooctanoate and preeclampsia in the C8 Health Project population. Environ. Res. 2016, 146 (1), 299–307. 10.1016/j.envres.2016.01.011. 26796985 PMC4761513

[ref22] Bartell S. M.; Johnson W. O. Estimating equations for biomarker based exposure estimation under non-steady-state conditions. Environ. Health 2011, 10 (1), 57 10.1186/1476-069X-10-57. 21668990 PMC3129579

[ref23] Fortin M. C.; Carrier G.; Bouchard M. Concentrations versus amounts of biomarkers in urine: A comparison of approaches to assess pyrethroid exposure. Environ. Health 2008, 7 (1), 55 10.1186/1476-069X-7-55. 18983658 PMC2615748

[ref24] Centers for Disease Control and Prevention (CDC), National Center for Health Statistics (NCHS). National Health and Nutrition Examination Survey. https://www.cdc.gov/nchs/nhanes/index.htm.

[ref25] United States Department of Agriculture (USDA). Pesticide Data Program (PDP, 2005–2009). www.ams.usda.gov/pdp.

[ref26] United States Environmental Protection Agency. Dietary Exposure Evaluation Model-Food Commodity Intake Database (DEEM-FCID) v4.02. https://www.epa.gov/pesticide-science-and-assessing-pesticide-risks/deem-fcidcalendex-software-installer.

[ref27] United States Department of Agriculture (USDA) Agricultural Research Service (ARS). What We Eat in America (WWEIA) 2022 https://www.ars.usda.gov/northeast-area/beltsville-md-bhnrc/beltsville-human-nutrition-research-center/food-surveys-research-group/docs/fndds/.

[ref28] United States Department of Agriculture. The Pesticide Data Program, 2022. https://www.usda.gov/.

[ref29] Models for Pesticide Risk Assessment. https://www.epa.gov/pesticide-science-and-assessing-pesticide-risks/models-pesticide-risk-assessment#deem.

[ref30] United States Environmental Protection Agency: Cyhalothrins -Acute and Average-Exposure Aggregate Dietary (Food, Drinking Water, Food Handling Establishment) Exposure and Risk Assessment in Support of Registration Review, 2017.

[ref31] Marsh J. R.; Woolen B. H.; Wilks M. F. The Metabolism and Pharmacokinetics of Lambda-Cyhalothrin in Man, 1994.

[ref32] Chester G.; Sabapathy N. N.; Woollen B. H. Exposure and health assessment during application of lambda-cyhalothrin for malaria vector control in Pakistan. Bull. World Health Organ. 1992, 70 (5), 615–619. 1464147 PMC2393370

[ref33] Khemiri R.; Côté J.; Fetoui H.; Bouchard M. Documenting the kinetic time course of lambda-cyhalothrin metabolites in orally exposed volunteers for the interpretation of biomonitoring data. Toxicol. Lett. 2017, 276 (May), 115–121. 10.1016/j.toxlet.2017.05.022. 28539253

[ref34] Mage D. T.; Allen R. H.; Gondy G.; Smith W.; Barr D. B.; Needham L. L. Estimating pesticide dose from urinary pesticide concentration data by creatinine correction in the Third National Health and Nutrition Examination Survey (NHANES-III). J. Exposure Anal. Environ. Epidemiol. 2004, 14 (6), 457–465. 10.1038/sj.jea.7500343. 15367927

[ref35] Mage D. T.; Allen R. H.; Kodali A. Creatinine corrections for estimating children’s and adult’s pesticide intake doses in equilibrium with urinary pesticide and creatinine concentrations. J. Exposure Sci. Environ. Epidemiol. 2008, 18 (4), 360–368. 10.1038/sj.jes.7500614. 17878925

[ref36] Blanker M. H.; Bohnen A. M.; Groeneveld F. P.; Bernsen R. M.; Prins A. D.; Ruud Bosch J. L. Normal Voiding Patterns and Determinants of Increased Diurnal and Nocturnal Voiding Frequency in Elderly Men. J. Urol. 2000, 164 (4), 1201–1205. 10.1016/S0022-5347(05)67141-8. 10992366

[ref37] Lukacz E. S.; Whitcomb E. L.; Lawrence J. M.; Nager C. W.; Luber K. M. Urinary frequency in community-dwelling women: what is normal?. Am. J. Obstet. Gynecol. 2009, 200 (5), 552.e1–552.e7. 10.1016/j.ajog.2008.11.006. PMC2695664 19249726

[ref38] Woollen B. H.; Marsh J. R.; Laird W. J. D.; Lesser J. E. The metabolism of cypermethrin in man: Differences in urinary metabolite profiles following oral and dermal administration. Xenobiotica 1992, 22 (8), 983–991. 10.3109/00498259209049904. 1413886

[ref39] Verrill L.; Lando A. M.; O’Connell K. M. Consumer vegetable and fruit washing practices in the United States, 2006 and 2010. Food Prot. Trends 2012, 32 (4), 164–172.

[ref40] Keikotlhaile B. M.; Spanoghe P.; Steurbaut W. Effects of food processing on pesticide residues in fruits and vegetables: A meta-analysis approach. Food Chem. Toxicol. 2010, 48 (1), 1–6. 10.1016/j.fct.2009.10.031. 19879312

[ref41] Georgopoulos P. G.; Sasso A. F.; Isukapalli S. S.; et al. Reconstructing population exposures to environmental chemicals from biomarkers. J. Exposure Sci. Environ. Epidemiol. 2009, 19, 149–171. 10.1038/jes.2008.9. PMC3068528 18368010

[ref42] Zhu Y.; Shin H.; Jiang L.; Bartell S. M. Retrospective exposure reconstruction using approximate Bayesian computation: A case study on perfluorooctanoic acid and preeclampsia. Environ. Res. 2022, 209, 112892 10.1016/j.envres.2022.112892. 35149111

[ref43] Turner B. M.; Van Zandt T. A tutorial on approximate Bayesian computation. J. Math. Psychol. 2012, 56 (2), 69–85. 10.1016/j.jmp.2012.02.005.

[ref44] CARES NG, 2023. https://caresng.org (accessed Jan).

[ref45] United States Geologival Survey (USGS), 2018 https://water.usgs.gov/nawqa/pnsp/usage/maps/compound_listing.php.

[ref46] Szarka A. Z.; Ramanarayanan T. S. Co-occurrence of Conazole Fungicide Residues in Raw Agricultural Commodities Sampled by the United States Department of Agriculture Pesticide Data Program. J. Agric. Food Chem. 2021, 69 (41), 12305–12313. 10.1021/acs.jafc.1c04062. 34633796

[ref47] United States Environmental Protection Agency. Lambda- & Gamma-Cyhalothrin: Human Health Draft Risk Assessment for Registration Review, 2017.

