# Supplementary material for: A Novel Approach to Integrate Human Biomonitoring Data with Model Predicted Dietary Exposures: A Crop Protection Chemical Case Study Using Lambda-Cyhalothrin
Source: J Agric Food Chem. 2024 May 8;72(20):11663–71. doi: 10.1021/acs.jafc.3c07071 (PMC11117394; doi:10.1021/acs.jafc.3c07071)
Supplement: Supplementary file 1 — jf3c07071_si_001.pdf [file jf3c07071_si_001.pdf]

Supporting Information

A Novel Approach to Integrate Human Biomonitoring Data with Model Predicted Dietary Exposures: A Crop Protection Chemical Case Study Using Lambda-Cyhalothrin

Nicholas Cuvelier[1, #], Raga Avanasi[2], Mark Grunenwald[2], Tharacad Ramanarayanan[2], Douglas C. Wolf[2], Scott M. Bartell[1,3*]

[1]Department of Environmental and Occupational Health, 856 Health Sciences Quad, Suite 3200 University of California, Irvine, CA 92617, United States

[2]Human Safety, Syngenta Crop Protection, LLC, Post Office Box 18300, Greensboro, North Carolina 27409, United States

[3]Department of Statistics and Department of Epidemiology and Biostatistics, University of California, Anteater Instruction Research Building, Suite 2030, Irvine, CA 92697, United States

[#] Author affiliation changed to California Department of Public Health at 1631 Alhambra Blvd, Suite 200 Sacramento, CA 95816, United States

Corresponding Author:

Scott Bartell

Email: sbartell@uci.edu

Validation of ADME Model

In order to determine if the ADME model was predicting realistic values for urinary 3PBA concentration, data obtained from a human controlled dosing trial was modeled and compared to the present model predictions and to the measured values[1]. For this validation, point estimates were used for the ADME parameters, rather than distributions and Monte Carlo simulation. The reported lambda cyhalothrin dose (5mg) was the initial dose at hour 0 in the model with a steady state dose of 0 for each participant. Although detailed dietary information was lacking for these participants, the administered dose was orders of magnitude greater than steady state doses implied by NHANES urinary concentrations, so it was expected that the impacts of any incidental dietary exposures were negligible in the controlled dosing trial. Results were plotted hour by hour, comparing predicted urinary 3PBA measurements to the actual urinary 3PBA measurements obtained during the trial. The predicted urinary measurements reasonably match the study measurements, with slight overprediction at the peak of the measured concentrations due to the assumption of instantaneous absorption and metabolism to 3PBA (Supplemental Figure 1). Around the timepoint corresponding to the peak of the measured urinary 3PBA the over prediction was between 8.5-13% for the log of the modelled vs log of the measured urinary concentrations. After this timepoint, the modelled urinary concentrations were still slightly above the measured values (6-11%). Despite some differences, all predictions are well within an order of magnitude of the measured values, and most remain extremely close throughout the follow-up time period for the trial.

While validating the initial model, a slightly more complex 2 compartment model was evaluated that included an additional gut compartment, to determine if this was a better fit for lambda-cyhalothrin (Supplemental Figure 1). To implement the 2 compartment model, a previously published closed form solution[2] for the serum concentration after a bolus dose at time 0 was adapted, using the same ADME parameter values as the 1 compartment model (with the i subscript removed to denote the use of

common point estimates rather than individualized values), but with an added first-order rate parameter a for absorption from the gut:

$$C_{i,t} = C_{i,0}e^{-kt} + fD_0a(e^{-kt}-e^{-at})/\ (a-kV)$$

The same bladder mixing and urinary excretion models were used for both the 1- and 2-compartment models. The 2-compartment model produced values that more often over-estimated urinary 3PBA, especially in the first 24 hours. These results suggest that the 1 compartment model is more suitable for predicting urinary 3PBA, so the 1 compartment model was used for the remainder of the study.

Supplemental Figure S1: Validation of Pharmacokinetic Model

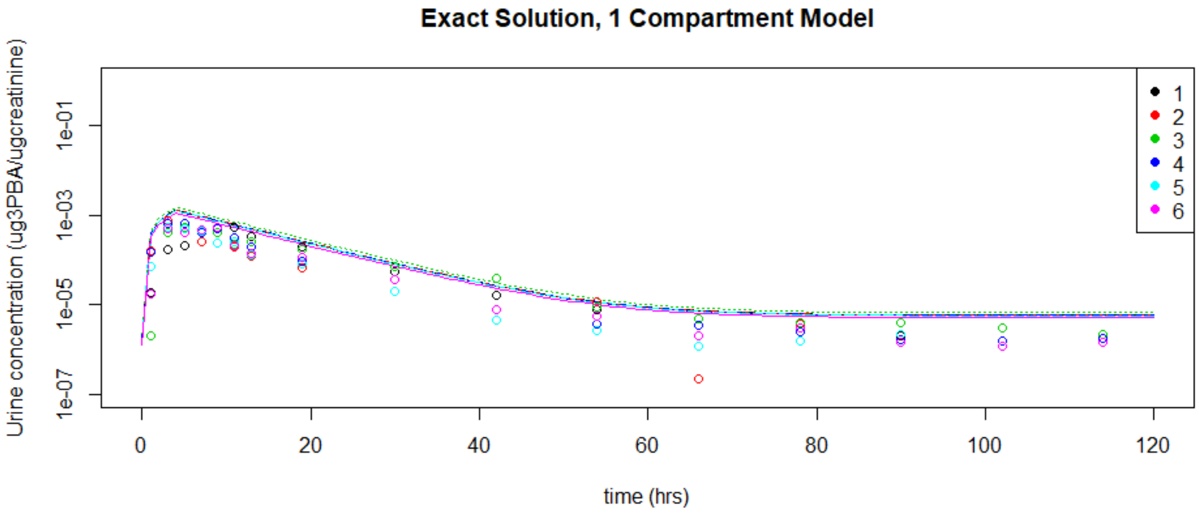

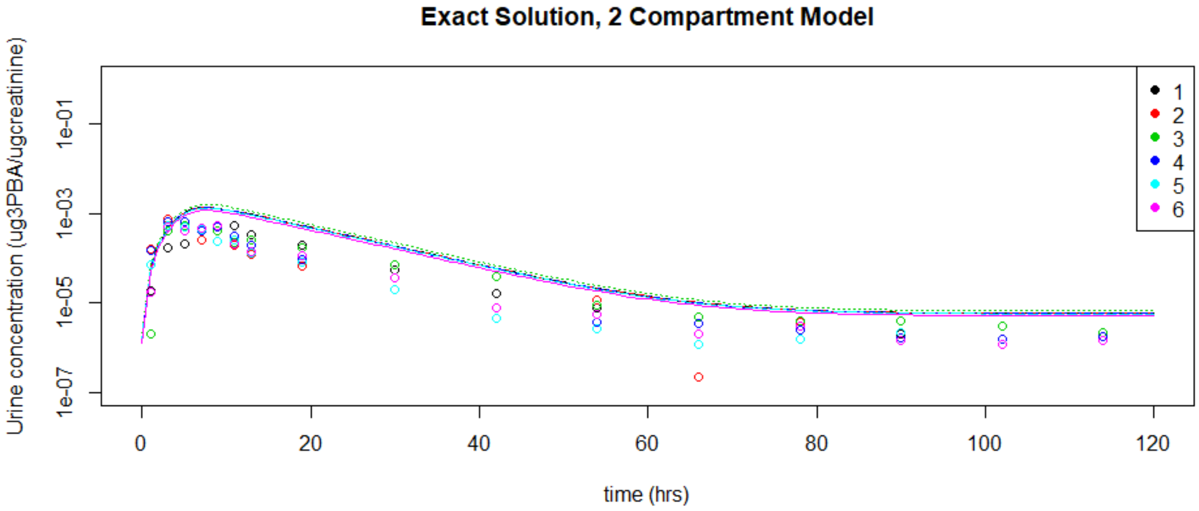

References

1. Marsh JR, Woolen BH, Wilks MF. The Metabolism and Pharmacokinetics of Lambda-Cyhalothrin in Man. 1994.

2. D'argenio DZ, Bae KS. Analytical solution of linear multi-compartment models with non-zero initial condition and its implementation with r. *Transl Clin Pharmacol*. 2019;27(2):43-51. doi:10.12793/tcp.2019.27.2.43

Supplemental Table S1. Model Predicted vs Measured Urinary 3PBA Values (µg3PBA/µgCreatinine)

With and Without ABC Thresholding

Percentiles	Measured urinary 3PBA no threshold	Model Predicted urinary 3PBA no threshold	Measured urinary 3PBA 1 degree of magnitude threshold	Model predicted urinary 3PBA 1 degree of magnitude threshold	Measured urinary 3PBA 0.5 degree of magnitude threshold	Model predicted urinary 3PBA 0.5 degree of magnitude threshold
Median	3.5E-07	3.8E-07	3.4E-07	4.3E-07	3.6E-07	4.2E-07
90th	2.5E-06	1.2E-06	1.6E-06	1.2E-06	1.2E-06	1.1E-06
95th	4.5E-06	1.6E-06	2.7E-06	1.5E-06	1.9E-06	1.5E-06
99th	1.5E-05	2.6E-06	5.2E-06	2.5E-06	3.6E-06	2.2E-06

Supplemental Figure S2. Histogram of Urinary 3PBA Residuals (log Predicted – log Observed) With and Without ABC Thresholding

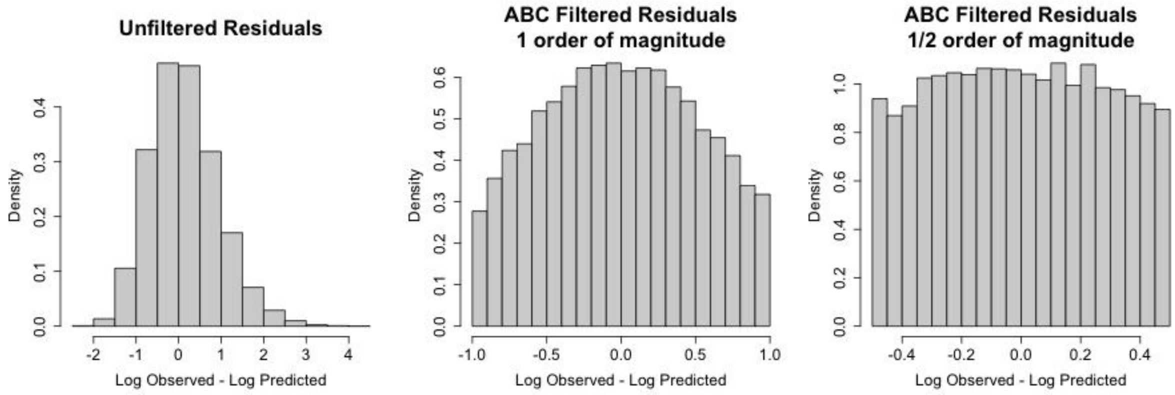

Supplemental R Code S1. run_all.R

```
### R code for running all code files to produce results for "A Novel Approach to Integrate
### Human Biomonitoring Data with Model Predicted Dietary Exposures: A Crop Protection
### Chemical Case Study Using Lambda-Cyhalothrin"
###
### Code by Nicholas Cuvelier and Scott M. Bartell
### University of California, Irvine
# set directory, RNG seed, options, and load libraries
setwd("Downloads") # set to preferred location for all user files
set.seed(200) # to ensure MC results are reproducible
library(Rlab)
library(nhanesA)
library(zoo)
library(survey)
library(chron)
library(dplyr)
library(survey)
options(timeout = max(1000, getOption("timeout")))
# execute all R code files in required order
source("load_nhanes.R") # load and process NHANES data
source("load_deem.R") # load and process DEEM data
source("compute_doses.R") # compute hourly doses for each participant
source("pk_model.R") # run PK model to predict urine concentrations
source("generate_results.R", echo=TRUE) # get summary statistics and plots
```

Supplemental R Code S2. load_deem.R

```
### R code for loading and processing DEEM output data for "A Novel Approach to Integrate
### Human Biomonitoring Data with Model Predicted Dietary Exposures: A Crop Protection
### Chemical Case Study Using Lambda-Cyhalothrin"
###
### Code by Nicholas Cuvelier and Scott M. Bartell
### University of California, Irvine
# The user must run DEEM manually first to create the input files loaded here.
# see https://www.epa.gov/sites/default/files/2015-09/documents/deem-user-guide-sep30-14.pdf
'child6to12' <- read.csv("child6to12.csv")
'child13to19' <- read.csv("child13to19.csv")
'adult20to49pt1' <- read.csv("adult20to49pt1.csv")
'adult20to49pt2' <- read.csv("adult20to49pt2.csv")
'adult20to49pt3' <- read.csv("adult20to49pt3.csv")
'adult50to99pt1' <- read.csv("adult50to99pt1.csv")
'adult50to99pt2' <- read.csv("adult50to99pt2.csv")
'adult50to99pt3' <- read.csv("adult50to99pt3.csv")
#Only keep relevant variables
child6to12 <- child6to12[, c("SEQN", "Day", "Sex", "Age", "Tot.Expos")]
child13to19 <- child13to19[, c("SEQN", "Day", "Sex", "Age", "Tot.Expos")]
adult20to49pt1 <- adult20to49pt1[, c("SEQN", "Day", "Sex", "Age", "Tot.Expos")]
adult20to49pt2 <- adult20to49pt2[, c("SEQN", "Day", "Sex", "Age", "Tot.Expos")]
adult20to49pt3 <- adult20to49pt3[, c("SEQN", "Day", "Sex", "Age", "Tot.Expos")]
adult50to99pt1 <- adult50to99pt1[, c("SEQN", "Day", "Sex", "Age", "Tot.Expos")]
adult50to99pt2 <- adult50to99pt2[, c("SEQN", "Day", "Sex", "Age", "Tot.Expos")]
adult50to99pt3 <- adult50to99pt3[, c("SEQN", "Day", "Sex", "Age", "Tot.Expos")]
#Remove blank rows
child6to12 <- child6to12[!is.na(child6to12$SEQN), ]
child13to19 <- child13to19[!is.na(child13to19$SEQN), ]
```

```
adult20to49pt1 <- adult20to49pt1[!is.na(adult20to49pt1$SEQN), ]

adult20to49pt2 <- adult20to49pt2[!is.na(adult20to49pt2$SEQN), ]

adult20to49pt3 <- adult20to49pt3[!is.na(adult20to49pt3$SEQN), ]

adult50to99pt1 <- adult50to99pt1[!is.na(adult50to99pt1$SEQN), ]

adult50to99pt2 <- adult50to99pt2[!is.na(adult50to99pt2$SEQN), ]

adult50to99pt3 <- adult50to99pt3[!is.na(adult50to99pt3$SEQN), ]

#Change variables to correct format

adult20to49 <- rbind(adult20to49pt1, adult20to49pt2, adult20to49pt3)

adult50to99 <- rbind(adult50to99pt1, adult50to99pt2, adult50to99pt3)

#Merge all subgroups into the total population

allpop <- rbind(child6to12, child13to19, adult20to49, adult50to99)

#Change format of variables

allpop$Tot.Expos <- as.numeric(as.character(allpop$Tot.Expos))

allpop$Day <- as.numeric(as.character(allpop$Day))

allpop$SEQN <- as.numeric(as.character(allpop$SEQN))

#Now remove the day 2 entries as PBA levels more reflective of day 1 intake

##child13to19_sub4 <- child13to19_sub3[child13to19_sub3$Day == 1, ]

#Instead of only keeping Day 1 here, keep both days to estimate steady state of 3PBA,

then remove Day 2 after

lambda_final <- merge(lambda_mealmerge3, allpop, by="SEQN")

# Remove those without a 3PBA result

lambda_tot <- lambda_final[!is.na(lambda_final$URXOPM), ]

#Remove those without creatinine result

lambda_tot <- lambda_tot[!is.na(lambda_tot$creatinine), ]

#Creatinine is is mg/dL, convert to ug/L to be consistent with 3PBA

lambda_tot$creatinineugL <- (lambda_tot$creatinine*10000)

#Now divide 3PBA by creatine to standardize (ug 3PBA per ug creatinine)

lambda_tot$PBAcreat <- (lambda_tot$URXOPM/lambda_tot$creatinineugL)
```

Supplemental R Code S3. load_nhanes.R

```
### R code for loading and processing NHANES data for "A Novel Approach to Integrate
### Human Biomonitoring Data with Model Predicted Dietary Exposures: A Crop Protection
### Chemical Case Study Using Lambda-Cyhalothrin"
###
### Code by Nicholas Cuvelier and Scott M. Bartell
### University of California, Irvine
#3-PBA are not available from 2003-2006 due to unnacceptable variance
##3PBA measurements below LOD are coded as 0.07 in NHANES data!!!!!
#Get 3-PBA diet, and demo data from 2007-2010
nhanesTables('LAB', 2007)
creat07 <- nhanes('ALB_CR_E')
lambda07 <- nhanes('UPHOPM_E')
sess07 <- nhanes('FASTQX_E')
nhanesTables('DEMO', 2007)
demo07 <- nhanes('DEMO_E')
nhanesTables('DIET', 2007)
diet07 <- nhanes('DR1IFF_E')
nhanesTables('EXAM', 2007)
exam07 <- nhanes('BMX_E')
nhanesTables('LAB', 2009)
creat09 <- nhanes('ALB_CR_F')
lambda09 <- nhanes('UPHOPM_F')
sess09 <- nhanes('FASTQX_F')
nhanesTables('DEMO', 2009)
demo09 <- nhanes('DEMO_F')
nhanesTables('DIET', 2009)
diet09 <- nhanes('DR1IFF_F')
nhanesTables('EXAM', 2009)
```

```r
exam09 <- nhanes('BMX_F')
#restrict pesticide and diet data to only lambda measurements
creat07_sub <- creat07[, c("SEQN", "URXUCR")]
lambda07_sub <- lambda07[, c("SEQN", "URXOPM")]
sess07_sub <- sess07[, c("SEQN", "PHDSESN", "PHAFSTHR")]
diet07_sub <- diet07[, c("SEQN", "DR1_020", "DR1_030Z")]
exam07_sub <- exam07[,c("SEQN", "BMXWT", "BMXHT")]
creat09_sub <- creat09[, c("SEQN", "URXUCR")]
lambda09_sub <- lambda09[, c("SEQN", "URXOPM")]
sess09_sub <- sess09[, c("SEQN", "PHDSESN", "PHAFSTHR")]
diet09_sub <- diet09[, c("SEQN", "DR1_020", "DR1_030Z")]
exam09_sub <- exam09[, c("SEQN", "BMXWT", "BMXHT")]
#Now merge DEMO and lab data by year
temp07 <- merge(demo07, lambda07_sub, by="SEQN")
temp07_2 <- merge(temp07, diet07_sub, by="SEQN")
temp07_3 <- merge(temp07_2, sess07_sub, by="SEQN")
temp07_4 <- merge(temp07_3, creat07_sub, by="SEQN")
full07 <- merge(temp07_4, exam07_sub, by="SEQN")
temp09 <- merge(demo09, lambda09_sub, by="SEQN")
temp09_2 <- merge(temp09, diet09_sub, by="SEQN")
temp09_3 <- merge(temp09_2, sess09_sub, by="SEQN")
temp09_4 <- merge(temp09_3, creat09_sub, by="SEQN")
full09 <- merge(temp09_4, exam09_sub, by="SEQN")
# Now need to remove duplicate rows
full07 <- unique(full07)
full09 <- unique(full09)
#Combine the dataframe
lambda_comb <- rbind(full07, full09)
lambda_comb$SEQN <- as.numeric(as.character(lambda_comb$SEQN))
```

```
#keep only the rows for the main mealtimes for each participant (1= breakfast 2=lunch
3=dinner, 4=supper, 10=desayuno 11=almuerzo 14=cena)
lambda_meal <- subset(lambda_comb, as.numeric(DR1_030Z) %in% c(1:4,10,11,14))
#extract numeric values for mealtimes
#hour values
lambda_meal$mealhr <- as.numeric(substr(lambda_meal$DR1_020,1,2))
#minute values
lambda_meal$mealmin <- as.numeric(substr(lambda_meal$DR1_020,4,5))
#divide minute values by 60 to get fractions of an hour
lambda_meal$mealmin <- lambda_meal$mealmin/60
#Then add the hours and minutes to get time of each meal occassion
lambda_meal$mealtime <- lambda_meal$mealhr + lambda_meal$mealmin
# Create new column for breakfast time only
lambda_meal$breakfast <- ifelse(as.numeric(lambda_meal$DR1_030Z) %in% c(1,10),
"breakfast", NA)
lambda_meal$lunch <- ifelse(as.numeric(lambda_meal$DR1_030Z) %in% c(2,11),
"lunch", NA)
lambda_meal$dinner <- ifelse(as.numeric(lambda_meal$DR1_030Z) %in% c(3,4,14),
"dinner", NA)
#Separate columns by eating occasion
lambda_breakfast <- lambda_meal[!is.na(lambda_meal$breakfast), ]
lambda_lunch <- lambda_meal[!is.na(lambda_meal$lunch), ]
lambda_dinner <- lambda_meal[!is.na(lambda_meal$dinner), ]
#Try to merge these DFs back, keeping all SEQNs and having columns for each eating
occasion
lambda_mealmerge <- merge(lambda_breakfast, lambda_lunch, by="SEQN", all=T)
lambda_mealmerge2 <- merge(lambda_mealmerge, lambda_dinner, by="SEQN",
all=T)
#Now try to only take unique IDs to remove duplicate entries
```

```
library(dplyr)
lambda_mealmerge3 <-
lambda_mealmerge2[!duplicated(lambda_mealmerge2$SEQN), ]
#Now clean up and remove extra columns
lambda_mealmerge3 <- lambda_mealmerge3[,
c(1,47:51,54,103:107,110,114:163,166,6,44,62,100, 41:43, 97:99)]
#Rename some of the columns
names(lambda_mealmerge3)[7] <- "breakfast"
names(lambda_mealmerge3)[13] <- "lunch"
names(lambda_mealmerge3)[64] <- "dinner"
#Reorder the columns so it makes sense
lambda_mealmerge3 <- lambda_mealmerge3[,
c(1,7,13,64,2:6,8:12,59:63,14:58,65:68,69:74)]
#Merge columns of interview sessions, fast times, height, and weight to remove NAs
lambda_mealmerge3$RIDAGEYR <-
coalesce(lambda_mealmerge3$RIDAGEYR.x,lambda_mealmerge3$RIDAGEYR.y,lam
bda_mealmerge3$RIDAGEYR)
lambda_mealmerge3$URXOPM <-
coalesce(lambda_mealmerge3$URXOPM.x,lambda_mealmerge3$URXOPM.y,lambda
_mealmerge3$URXOPM)
lambda_mealmerge3$session <-
coalesce(lambda_mealmerge3$PHDSESN.x,lambda_mealmerge3$PHDSESN.y,lamb
da_mealmerge3$PHDSESN)
lambda_mealmerge3$fast <-
coalesce(lambda_mealmerge3$PHAFSTHR.x,lambda_mealmerge3$PHAFSTHR.y,la
mbda_mealmerge3$PHAFSTHR)
lambda_mealmerge3$weight <- coalesce(lambda_mealmerge3$BMXWT.x,
lambda_mealmerge3$BMXWT.y, lambda_mealmerge3$BMXWT)
lambda_mealmerge3$height <- coalesce(lambda_mealmerge3$BMXHT.x,
```

```
lambda_mealmerge3$BMXHT.y, lambda_mealmerge3$BMXHT)
lambda_mealmerge3$creatinine <- coalesce(lambda_mealmerge3$URXUCR.x,
lambda_mealmerge3$URXUCR.y, lambda_mealmerge3$URXUCR)
lambda_mealmerge3$SDMVPSU <- coalesce(lambda_mealmerge3$SDMVPSU.x,
lambda_mealmerge3$SDMVPSU.y, lambda_mealmerge3$SDMVPSU)
lambda_mealmerge3$SDMVSTRA <- coalesce(lambda_mealmerge3$SDMVSTRA.x,
lambda_mealmerge3$SDMVSTRA.y, lambda_mealmerge3$SDMVSTRA)
lambda_mealmerge3$WTMEC2YR <- coalesce(lambda_mealmerge3$WTMEC2YR.x,
lambda_mealmerge3$WTMEC2YR.y, lambda_mealmerge3$WTMEC2YR)
##Note, there are some NAs in height and weight which will lead to NAs in creatinine
clearance calculations
#Remove extra URXOPM and RIDAGEYR columns to be consistent with previous code
in terms of # of columns
lambda_mealmerge3 <- lambda_mealmerge3[, c(1:64, 75:79)]
#Now create columnswith 1 or 0 for whether or not each meal was eaten, then add
results
lambda_mealmerge3$meal1 <- ifelse(lambda_mealmerge3$breakfast != "NA", 1, 0)
lambda_mealmerge3$meal2 <- ifelse(lambda_mealmerge3$lunch != "NA", 1, 0)
lambda_mealmerge3$meal3 <- ifelse(lambda_mealmerge3$dinner != "NA", 1, 0)
#Replace NAs with 0s
lambda_mealmerge3$meal1[is.na(lambda_mealmerge3$meal1)] <- 0
lambda_mealmerge3$meal2[is.na(lambda_mealmerge3$meal2)] <- 0
lambda_mealmerge3$meal3[is.na(lambda_mealmerge3$meal3)] <- 0
#Sum meal occasions to determine total number of meals per SEQN
lambda_mealmerge3$nummeals <- (lambda_mealmerge3$meal1 +
lambda_mealmerge3$meal2 + lambda_mealmerge3$meal3)
#Finally, reorder the columns
lambda_mealmerge3 <- lambda_mealmerge3[, c(1:9,73,67,10:66, 68:72)]
```

Supplemental R Code S4. pk_model.R

```
### R code for running PK model to produce results for "A Novel Approach to Integrate
### Human Biomonitoring Data with Model Predicted Dietary Exposures: A Crop Protection
### Chemical Case Study Using Lambda-Cyhalothrin"
### Code by Nicholas Cuvelier and Scott M. Bartell
### University of California, Irvine
# Generate random variates for PK parameters
lambda_tot4$hl <- rgamma(24.23668639, 3.786982249, n=21035)
lambda_tot4$percent_3PBA <- rbeta(11.119, 31.357, n=21035)
lambda_tot4$V <- rgamma(6.775302768, 0.382785467, n=21035)
lambda_tot4$rem4oh <- rbeta(7.757, 5.864305, n=21035)
# PK MODEL!
# loop through each participant
for (i in 1:length(lambda_tot4$SEQN)) {
hl_pk = lambda_tot4$hl[i] # per hour, 6.4 ± 1.3 from plasma, 5.9 ± 1.4 from urine
w = dbern(1, 0.5) # washing behavior and following pesticide reduction
r = rbeta(1, 3.917, 7.806333)
wash = 1-(w*r)
k=log(2)/hl_pk
percent_3PBA_pk <- lambda_tot4$percent_3PBA[i]
V_pk = lambda_tot4$V[i] #17.7 ± 6.8 L (Khemiri et al, 2017)
t = 48 #t in hours
D = rep(0,t)# 0 dose in most hours
D[lambda_tot4$breakfast[i]] = lambda_tot4$Tot.Expos_mgkg[i] /
lambda_tot4$nummeals[i]
D[lambda_tot4$lunch[i]] = lambda_tot4$Tot.Expos_mgkg[i] / lambda_tot4$nummeals[i]
D[lambda_tot4$dinner[i]] = lambda_tot4$Tot.Expos_mgkg[i] /
lambda_tot4$nummeals[i]
lambda_tot4$breakfast2[i] <- lambda_tot4$breakfast[i] + 24
```

```
lambda_tot4$lunch2[i] <- lambda_tot4$lunch[i] + 24
lambda_tot4$dinner2[i] <- lambda_tot4$dinner[i] + 24
if(!is.na(lambda_tot4$breakfast2[i]) && (lambda_tot4$turinecoll[i] -
lambda_tot4$breakfast2[i]) > lambda_tot4$fast[i]) {
D[lambda_tot4$breakfast2[i]] <- D[lambda_tot4$breakfast[i]]
}
#Lunch day of urine collection. Need to account for possibly breakfast and lunch
if(!is.na(lambda_tot4$lunch2[i]) && (lambda_tot4$turinecoll[i] - lambda_tot4$lunch2[i])
> lambda_tot4$fast[i]) {
D[lambda_tot4$lunch2[i]] <- D[lambda_tot4$lunch[i]]
}
#Dinner day of urine collection. Need to account for possibly breakfast, lunch and
dinner
if(!is.na(lambda_tot4$dinner2[i]) && (lambda_tot4$turinecoll[i] -
lambda_tot4$dinner2[i]) > lambda_tot4$fast[i]) {
D[lambda_tot4$dinner2[i]] <- D[lambda_tot4$dinner[i]]
}
#Residential lambda exposure estimation
#Start by calculating residential dose for day 1 and day 2
#Multiply by bodyweight to get mg/day from mg/kg/day
#Add for loops for each IF statement and [i] for all variables
#Need a day 1 and day 2 dosage
#Make dose into hour by hour, divide by 24 for mg/hour
#Add this to each position of dietary dosage vector: D+residential value per hour
#Use [i] on all variable except D
#cHANGE THE VALUE BASED ON AGE FROM rAGA epa DOCUMENT
if(lambda_tot4$RIDAGEYR[i] <16){
lambda_tot4$residential_day1[i] <- 0.013*lambda_tot4$weight[i]*resdose
}
```

```
if(lambda_tot4$RIDAGEYR[i] >= 16){
lambda_tot4$residential_day1[i] <- 0.035*lambda_tot4$weight[i]*resdose
}
D <- (D*wash) + (lambda_tot4$residential_day1[i]/24)
# check to see if anybody is skipping meals
# are participants fasting for entire day of MEC visit? If yes, D is done. If no, should fill
in possible dose from consumption on current day.
# hour by hour according to dietary intake (may need to lump into typical meal times)
Cserum[i, 1] = lambda_tot4$SS_mgkg[i]/(24*k*V_pk) # need to enter an
estimated starting non-zero serum concentration here, for start of day 1
#Also multiply k by 24 to change units to days to match the dosage
# loop through each hour, adding new dose and exponential decay from previous
hour's serum
for (j in 1:t) {
Cserum[i, j+1] = Cserum[i, j]*exp(-k) + D[j]*percent_3PBA_pk/k/V_pk*(1-exp(-k))
rem4oh_pk <- lambda_tot4$rem4oh[i]
turine <- lambda_tot4$turinecoll[i]
#To get urinary concentration, use Cserum*V*kMU. Units should become
mg(3PBA)/hour
kMU <- k
cncl <- lambda_tot4$cnclhr[i]
Curine[i, ] <- (Cserum[i, ]*V_pk*kMU*1000*rem4oh_pk)/cncl
void <- sample(4:6, 1)
Curine_pad <- c(rep(Curine[i, void], void-1), Curine[i, ])
#Now try rollmean function in zoo package
t_k <- lambda_tot4$turinecoll[i]
lambda_tot4$Curine3[i] <- mean(Curine[i, t_k:(t_k-void+1)])
} # end time loop
} # end participant loop
```

Supplemental R Code S5. compute_doses.R

```
### R code for computing doses for each participant in "A Novel Approach to Integrate
### Human Biomonitoring Data with Model Predicted Dietary Exposures: A Crop Protection
### Chemical Case Study Using Lambda-Cyhalothrin"
###
### Code by Nicholas Cuvelier and Scott M. Bartell
### University of California, Irvine
#Reorder columns again
lambda_tot2 <- lambda_tot[, c(1:11,75:79,12:74)]
#Now take the mean of Tot.Expos for each person across both days
lambda_tot2$SS <- with(lambda_tot2, ave(Tot.Expos, SEQN, FUN=mean))
#Reorder columns once again
lambda_tot2 <- lambda_tot2[, c(1:11,80,75:79,12:74)]
#Now remove the day 2 entries so we only use Day 1 values in the PK model
lambda_tot2 <- lambda_tot2[lambda_tot2$Day == 1, ]
# Remove those missing height or weight measurements
lambda_tot2 <- lambda_tot2[!is.na(lambda_tot2$weight), ]
#Convert Tot.Expos from mg/kg/day to mg/day by multiplying by weight
lambda_tot2$Tot.Expos_mgkg <- lambda_tot2$Tot.Expos*lambda_tot2$weight
#Also convert the steady state exposure dose to mg/day from mg/kg/day for unit
conversion in PK model
lambda_tot2$SS_mgkg <- lambda_tot2$SS*lambda_tot2$weight
#Add in variable resdose for Boolean logic to add or remove residential doses of
lambda
resdose=F
#Add in residential dose from EPA to SS measurements
lambda_tot2$SS_mgkg[lambda_tot2$RIDAGEYR < 16] <-
lambda_tot2$SS_mgkg[lambda_tot2$RIDAGEYR < 16] +
((0.013*lambda_tot2$weight[lambda_tot2$RIDAGEYR < 16])*resdose)
```

```
lambda_tot2$SS_mgkg[lambda_tot2$RIDAGEYR >= 16] <-
lambda_tot2$SS_mgkg[lambda_tot2$RIDAGEYR >= 16] +
((0.035*lambda_tot2$weight[lambda_tot2$RIDAGEYR >= 16])*resdose)
### Now create time variable for time of urine collection
#Have to separate dataframe by session time first, merge back later
lambda_turine1 <- lambda_tot2[as.numeric(lambda_tot2$session) == 1, ]
lambda_turine2 <- lambda_tot2[as.numeric(lambda_tot2$session) == 2, ]
lambda_turine3 <- lambda_tot2[as.numeric(lambda_tot2$session) == 3, ]
#create time column for lambda_urine1 first, then MC sampling
hm <- merge(8.5:12, seq(0, 59, by = 1))
hm1 <- chron(time = paste(hm$x, ':', hm$y, ':', 0))
lambda_turine1$turine <- sample(hm1, size=length(lambda_turine1$SEQN),
replace=T) # MC sampling of exact times
summary(lambda_turine1$turine)
hm2 <- merge(12.5:17, seq(0, 59, by = 1))
hm3 <- chron(time = paste(hm2$x, ':', hm2$y, ':', 0))
lambda_turine2$turine <- sample(hm3, size=length(lambda_turine2$SEQN),
replace=T) # MC sampling of exact times
summary(lambda_turine2$turine)
hm4 <- merge(17.5:21, seq(0, 59, by = 1))
hm5 <- chron(time = paste(hm4$x, ':', hm4$y, ':', 0))
lambda_turine3$turine <- sample(hm5, size=length(lambda_turine3$SEQN),
replace=T) # MC sampling of exact times
#Combine the dataframes again
lambda_tot3 <- merge(lambda_turine1, lambda_turine2, all=T, no.dupes=T)
lambda_tot4 <- merge(lambda_tot3, lambda_turine3, all=T, no.dupes=T)
#Reorder columns of lambda_tot4
lambda_tot4 <- lambda_tot4[, c(1:12, 20:22, 81:83, 13:19, 23:80)]
#Change time of urine collection variable to numeric
```

```
lambda_tot4$urinehr <- as.numeric(substr(lambda_tot4$turine,1,2))
lambda_tot4$urinemin <- as.numeric(substr(lambda_tot4$turine,4,5))
#Change mins into fractions of an hour
lambda_tot4$urinemin <- lambda_tot4$urinemin/60
#Add hours and mins to get ttime in numeric
lambda_tot4$turinenum <- lambda_tot4$urinehr + lambda_tot4$urinemin
#reorganize columns
lambda_tot4 <- lambda_tot4[, c(1:18, 86, 19:85 )]
# ROUND MEALTIMES AND URINE COLLECTION TIMES
# Use ceiling to round up to next hour, add .01 hours so nobody starts at 0 for mealtime
lambda_tot4$breakfast <- ceiling(lambda_tot4$breakfast+0.01)
lambda_tot4$lunch <- ceiling(lambda_tot4$lunch+0.01)
lambda_tot4$dinner <- ceiling(lambda_tot4$dinner+0.01)
lambda_tot4$turinenum <- ceiling(lambda_tot4$turinenum+0.01)
lambda_tot4$turinecoll <- lambda_tot4$turinenum + 24
# Now to add in creatinine variable for standardization in PK model, get ug/day for
answer (from Mage et al, 2004)
# Convert to mg/day to be consistent with children formulas, then convert back to
ug/day after
lambda_tot4$cncl <- ifelse(lambda_tot4$Sex == "M",
(1.93*(140-lambda_tot4$RIDAGEYR)*((lambda_tot4$weight)^1.5)
*((lambda_tot4$height)^0.5))/1000,
(1.64*(140-lambda_tot4$RIDAGEYR)*((lambda_tot4$weight)^1.5)*((lambda_tot4$heig
ht)^0.5))/1000)
lambda_tot4$cncl <- ifelse(lambda_tot4$Sex == "M" & lambda_tot4$RIDAGEYR <18 &
lambda_tot4$height < 168,
lambda_tot4$height*(6.265 + 0.0564*(lambda_tot4$height-168)),
lambda_tot4$cncl)
lambda_tot4$cncl <- ifelse(lambda_tot4$Sex == "M" & lambda_tot4$RIDAGEYR <18 &
```

```
lambda_tot4$height >= 168,
lambda_tot4$height*(6.265 + 0.2550*(lambda_tot4$height-168)),
lambda_tot4$cncl)
lambda_tot4$cncl <- ifelse(lambda_tot4$Sex == "F" & lambda_tot4$RIDAGEYR <18,
2.045*(e(0.01552*(lambda_tot4$height-90))), lambda_tot4$cncl)
#Convert back to ug/day
lambda_tot4$cncl <- lambda_tot4$cncl*1000
#Now divide by 24 to get ug/hour
lambda_tot4$cnclhr <- lambda_tot4$cncl/24
lambda_tot4 <- lambda_tot4[!is.na(lambda_tot4$cnclhr), ]
#Add in empty columns for breakfast2, lunch2, dinner2
lambda_tot4$breakfast2 <- NA
lambda_tot4$lunch2 <- NA
lambda_tot4$dinner2 <- NA
lambda_tot4$residential_day1 <- NA
lambda_tot4$CEurine <- NA
lambda_tot4$CEurine_ug <- NA
#Make fasting NAs into 0
lambda_tot4$fast[is.na(lambda_tot4$fast)] <- 0
#2 people had time 0 listed for dinner, meant midnight? but which day??? Could also
make it 1
lambda_tot4$dinner[lambda_tot4$dinner == 0] <- 24
# prepare a vector to store results of serum calculations
Cserum = matrix(NA,length(lambda_tot4$SEQN), 49)
Curine = matrix(NA,length(lambda_tot4$SEQN), 49)
```

Supplemental R Code S6. generate_results.R

```
### R code for results (summary statistics and plots) for "A Novel Approach to Integrate
### Human Biomonitoring Data with Model Predicted Dietary Exposures: A Crop Protection
### Chemical Case Study Using Lambda-Cyhalothrin"
###
### Code by Nicholas Cuvelier and Scott M. Bartell
### University of California, Irvine
# Determine ABC thresholds (TRUE for accept, FALSE for reject)
lambda_tot4$Curine_acc <- (log10(lambda_tot4$Curine3) <
1+(1*log10(lambda_tot4$PBAcreat))) &
(log10(lambda_tot4$Curine3) > -1+(1*log10(lambda_tot4$PBAcreat)))
lambda_tot4$Curine_acc2 <- (log10(lambda_tot4$Curine3) <
0.5+(1*log10(lambda_tot4$PBAcreat))) &
(log10(lambda_tot4$Curine3) > -0.5+(1*log10(lambda_tot4$PBAcreat)))
# Plot log10 scale predicted vs. observed, with equivalence line (slope 0)
# and threshold lines at +/- 1 (or 1/2) order of magnitude difference
# 3 graphs side by side in same window
par(mfrow = c(1, 3))
plot(log10(lambda_tot4$PBAcreat), log10(lambda_tot4$Curine3), ylab="Model
predicted urinary 3PBA (ug3PBA/ugCreatinine)",
xlab= "NHANES measured urinary 3PBA (ug3PBA/ugCreatinine)", xlim=c(-8,-4),
ylim=c(-8,-4), abline(0,1), cex=0.5, pch=".",
main="Predicted vs Measured Urinary 3PBA (Log10 scale)")
plot(log10(lambda_tot4$PBAcreat), log10(lambda_tot4$Curine3), ylab="Model
predicted urinary 3PBA (ug3PBA/ugCreatinine)",
xlab= "NHANES measured urinary 3PBA (ug3PBA/ugCreatinine)", xlim=c(-8,-4),
ylim=c(-8,-4), abline(0,1), cex=0.5, pch=".",
main="Predicted vs Measured Urinary 3PBA (Log10 scale)",
col=2-lambda_tot4$Curine_acc)
```

```
abline(1, 1, lty=2, col=2)
abline(-1, 1, lty=2, col=2)
plot(log10(lambda_tot4$PBAcreat), log10(lambda_tot4$Curine3), ylab="Model
predicted urinary 3PBA (ug3PBA/ugCreatinine)",
xlab= "NHANES measured urinary 3PBA (ug3PBA/ugCreatinine)", xlim=c(-8,-4),
ylim=c(-8,-4), abline(0,1), cex=0.5, pch=".",
main="Predicted vs Measured Urinary 3PBA (Log10 scale)",
col=2-lambda_tot4$Curine_acc2)
abline(0.5, 1, lty=2, col=2)
abline(-0.5, 1, lty=2, col=2)
# Plot residual histograms, log10 scale
jpeg(width=900,height=300)
par(mfrow = c(1, 3))
hist(log10(lambda_tot4$PBAcreat)-log10(lambda_tot4$Curine3), main="Unfiltered
Residuals",
xlab = "Log Observed - Log Predicted", freq=F, cex.lab=1.5, cex.axis=1.5, cex.main=2)
hist((log10(lambda_tot4$PBAcreat)-log10(lambda_tot4$Curine3))[lambda_tot4$Curine
_acc], main="ABC Filtered Residuals\n1 order of magnitude",
xlab = "Log Observed - Log Predicted", freq=F, cex.lab=1.5, cex.axis=1.5, cex.main=2)
hist((log10(lambda_tot4$PBAcreat)-log10(lambda_tot4$Curine3))[lambda_tot4$Curine
_acc2] , main="ABC Filtered Residuals\n1/2 order of magnitude",
xlab = "Log Observed - Log Predicted", freq=F, cex.lab=1.5, cex.axis=1.5, cex.main=2)
dev.off()
# predicted vs. observed correlations and p-values
cor.test(lambda_tot4$PBAcreat, lambda_tot4$Curine3)
cor.test(lambda_tot4$PBAcreat[lambda_tot4$Curine_acc],
lambda_tot4$Curine3[lambda_tot4$Curine_acc])
cor.test(lambda_tot4$PBAcreat[lambda_tot4$Curine_acc2],
lambda_tot4$Curine3[lambda_tot4$Curine_acc2])
```

```
#How many points are retained after ABC thresholding?
sum(lambda_tot4$Curine3[lambda_tot4$Curine_acc] != "FALSE")/21035
sum(lambda_tot4$Curine3[lambda_tot4$Curine_acc2] != "FALSE")/21035
#Get the quantiles for each group (unweighted)
quantile(log10(lambda_tot4$PBAcreat), probs=c(.01, .05, .1, .5, .9, .95, .99))
quantile(log10(lambda_tot4$Curine3), probs=c(.01, .05, .1, .5, .9, .95, .99))
quantile(log10(lambda_tot4$Curine3[lambda_tot4$Curine_acc]), probs=c(.01, .05, .1,
.5, .9, .95, .99))
quantile(log10(lambda_tot4$Curine3[lambda_tot4$Curine_acc2]), probs=c(.01, .05, .1,
.5, .9, .95, .99))
quantile(lambda_tot4$PBAcreat, probs=c(.01, .05, .1, .5, .9, .95, .99))
quantile(lambda_tot4$PBAcreat[lambda_tot4$Curine_acc], probs=c(.01, .05, .1, .5, .9,
.95, .99))
quantile(lambda_tot4$PBAcreat[lambda_tot4$Curine_acc2], probs=c(.01, .05, .1, .5, .9,
.95, .99))
quantile(lambda_tot4$Curine3, probs=c(.01, .05, .1, .5, .9, .95, .99))
quantile(lambda_tot4$Curine3[lambda_tot4$Curine_acc], probs=c(.01, .05, .1, .5, .9,
.95, .99))
quantile(lambda_tot4$Curine3[lambda_tot4$Curine_acc2], probs=c(.01, .05, .1, .5, .9,
.95, .99))
mean(Cserum[, 1])
#Check the original dose rates of lambda (mg/kg/day) compared to those after ABC
quantile(lambda_tot4$Tot.Expos, probs=c(.01, .05, .1, .5, .9, .95, .99))
quantile(lambda_tot4$Tot.Expos[lambda_tot4$Curine_acc], probs=c(.01, .05, .1, .5, .9,
.95, .99))
quantile(lambda_tot4$Tot.Expos[lambda_tot4$Curine_acc2], probs=c(.01, .05, .1, .5,
.9, .95, .99))
### Now do analysis using survey weights
# Average values across non-unique SEQN so we have 1 entry per person
```

```
lambda_tot4$weight4yr <- 1/2*lambda_tot4$WTMEC2YR
## unfiltered
lambda_tot4weight <- lambda_tot4 %>%
group_by(SEQN) %>%
summarise(Curine3 = mean(Curine3),
PBAcreat = mean(PBAcreat),
Tot.Expos = mean(Tot.Expos),
weight4yr = weight4yr,
SDMVPSU = SDMVPSU,
SDMVSTRA = SDMVSTRA,
Curine_acc = Curine_acc,
Curine_acc2 = Curine_acc2,
SEQN = SEQN)
lambda_tot4weight2 <- unique(lambda_tot4weight)
lambda_tot4analysis <- lambda_tot4weight2 %>% select(
SDMVPSU,
SDMVSTRA,
weight4yr,
Curine3,
PBAcreat,
Tot.Expos,
SEQN
)
lambda_tot4design <- svydesign( id = ~SDMVPSU,
strata = ~SDMVSTRA,
weights = ~weight4yr,
nest = TRUE,
data = lambda_tot4analysis)
svyquantile(~Curine3, design=lambda_tot4design, c(0.5,0.9,0.95,0.99), na.rm=T)
```

```
svyquantile(~PBAcreat, design=lambda_tot4design, c(0.5,0.9,0.95,0.99), na.rm=T)
svyquantile(~Tot.Expos, design=lambda_tot4design, c(0.5,0.9,0.95,0.99), na.rm=T)
#Correlations
var <- svyvar(~PBAcreat + Curine3, design = lambda_tot4design)
var <- as.matrix(var)
cor <- cov2cor(var)
cor
### include thresholding for weighted analysis
# need to accept/reject before averaging within each SEQN (i.e., may accept some but
not all iterations for each person)
## filtered, within 1 order of magnitude
lambda_tot5 <- lambda_tot4[lambda_tot4$Curine_acc == T, ]
lambda_tot5weight <- lambda_tot5 %>%
group_by(SEQN) %>%
summarise(Curine3 = mean(Curine3),
PBAcreat = mean(PBAcreat),
Tot.Expos = mean(Tot.Expos),
weight4yr = weight4yr,
SDMVPSU = SDMVPSU,
SDMVSTRA = SDMVSTRA,
Curine_acc = Curine_acc,
Curine_acc2 = Curine_acc2,
SEQN = SEQN)
lambda_tot5weight2 <- unique(lambda_tot5weight)
lambda_tot5analysis <- lambda_tot5weight %>% select(
SDMVPSU,
SDMVSTRA,
weight4yr,
Curine3,
```

```
PBAcreat,
Tot.Expos,
SEQN
)
lambda_tot5design <- svydesign( id = ~SDMVPSU,
strata = ~SDMVSTRA,
weights = ~weight4yr,
nest = TRUE,
data = lambda_tot5analysis)
svyquantile(~Curine3, design=lambda_tot5design, c(0.5,0.9,0.95,0.99), na.rm=T)
svyquantile(~PBAcreat, design=lambda_tot5design, c(0.5,0.9,0.95,0.99), na.rm=T)
svyquantile(~Tot.Expos, design=lambda_tot5design, c(0.5,0.9,0.95,0.99), na.rm=T)
#Correlations
var2 <- svyvar(~PBAcreat + Curine3, design = lambda_tot5design)
var2 <- as.matrix(var2)
cor <- cov2cor(var2)
cor
## filtered, within 1/2 order of magnitude
lambda_tot6 <- lambda_tot4[lambda_tot4$Curine_acc2 == T, ]
lambda_tot6weight <- lambda_tot6 %>%
group_by(SEQN) %>%
summarise(Curine3 = mean(Curine3),
PBAcreat = mean(PBAcreat),
Tot.Expos = mean(Tot.Expos),
weight4yr = weight4yr,
SDMVPSU = SDMVPSU,
SDMVSTRA = SDMVSTRA,
Curine_acc = Curine_acc,
Curine_acc2 = Curine_acc2,
```

```
SEQN = SEQN)
lambda_tot6weight2 <- unique(lambda_tot6weight)
lambda_tot6analysis <- lambda_tot6weight %>% select(
SDMVPSU,
SDMVSTRA,
weight4yr,
Curine3,
PBAcreat,
Tot.Expos,
SEQN
)
lambda_tot6design <- svydesign( id = ~SDMVPSU,
strata = ~SDMVSTRA,
weights = ~weight4yr,
nest = TRUE,
data = lambda_tot6analysis)
svyquantile(~Curine3, design=lambda_tot6design, c(0.5,0.9,0.95,0.99), na.rm=T)
svyquantile(~PBAcreat, design=lambda_tot6design, c(0.5,0.9,0.95,0.99), na.rm=T)
svyquantile(~Tot.Expos, design=lambda_tot6design, c(0.5,0.9,0.95,0.99), na.rm=T)
#Correlations
var3 <- svyvar(~PBAcreat + Curine3, design = lambda_tot6design)
var3 <- as.matrix(var3)
cor <- cov2cor(var3)
cor
```
